# Test–retest reliability of a finger‐tapping fMRI task in a healthy population

**DOI:** 10.1111/ejn.15865

**Published:** 2022-11-25

**Authors:** Florian Wüthrich, Stephanie Lefebvre, Niluja Nadesalingam, Jessica A. Bernard, Vijay A. Mittal, Stewart A. Shankman, Sebastian Walther

**Affiliations:** ^1^ Translational Research Center, University Hospital of Psychiatry and Psychotherapy University of Bern Bern Switzerland; ^2^ Translational Imaging Center (TIC) Swiss Institute for Translational and Entrepreneurial Medicine Bern Switzerland; ^3^ Graduate School for Health Sciences University of Bern Bern Switzerland; ^4^ Department of Psychological and Brain Sciences, Texas A&M Institute for Neuroscience Texas A&M University College Station Texas USA; ^5^ Department of Psychiatry and Behavioral Sciences Northwestern University Chicago Illinois USA; ^6^ Department of Psychology Northwestern University Evanston Illinois USA; ^7^ Institute for Innovations in Developmental Sciences Northwestern University Evanston/Chicago Illinois USA; ^8^ Institute for Policy Research Northwestern University Evanston Illinois USA; ^9^ Medical Social Sciences Northwestern University Chicago Illinois USA

**Keywords:** Dice similarity coefficient, DSC, ICC, intraclass correlation coefficient, motor task

## Abstract

Measuring brain activity during functional MRI (fMRI) tasks is one of the main tools to identify brain biomarkers of disease or neural substrates associated with specific symptoms. However, identifying correct biomarkers relies on reliable measures. Recently, poor reliability was reported for task‐based fMRI measures. The present study aimed to demonstrate the reliability of a finger‐tapping fMRI task across two sessions in healthy participants. Thirty‐one right‐handed healthy participants aged 18–60 years took part in two MRI sessions 3 weeks apart during which we acquired finger‐tapping task‐fMRI. We examined the overlap of activations between sessions using Dice similarity coefficients, assessing their location and extent. Then, we compared amplitudes calculating intraclass correlation coefficients (ICCs) in three sets of regions of interest (ROIs) in the motor network: literature‐based ROIs (10‐mm‐radius spheres centred on peaks of an activation likelihood estimation), anatomical ROIs (regions as defined in an atlas) and ROIs based on conjunction analyses (superthreshold voxels in both sessions). Finger tapping consistently activated expected regions, for example, left primary sensorimotor cortices, premotor area and right cerebellum. We found good‐to‐excellent overlap of activations for most contrasts (Dice coefficients: .54–.82). Across time, ICCs showed large variability in all ROI sets (.04–.91). However, ICCs in most ROIs indicated fair‐to‐good reliability (mean = .52). The least specific contrast consistently yielded the best reliability. Overall, the finger‐tapping task showed good spatial overlap and fair reliability of amplitudes on group level. Although caution is warranted in interpreting correlations of activations with other variables, identification of activated regions in response to a task and their between‐group comparisons are still valid and important modes of analysis in neuroimaging to find population tendencies and differences.

## Graphical abstract

This study reports on the group‐level test–retest reliability of a finger‐tapping fMRI task. We examined overlap of activations across two sessions using Dice similarity coefficients and investigated amplitudes of activations by calculation of region of interest (ROI)‐based intraclass correlation coefficients in three sets of ROIs. The task included four different tapping conditions, and 31 healthy adults were included in the analyses. We found good‐to‐excellent overlap and fair‐to‐good amplitude agreement in most contrasts and ROI sets.

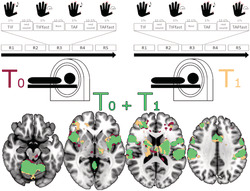

**Abbreviations**
ALE: activation likelihood estimation
BOLD: blood oxygen level‐dependent
CI: confidence interval
COVID‐19: coronavirus disease 2019
DSC: Dice similarity coefficient
fMRI: functional MRI
FOV: field of view
FWE: family‐wise error rate
FWHM: full width at half maximum
ICC: intraclass correlation coefficient
M1: primary motor cortex
mbep2d: multiband accelerated echo planar imaging sequence
MP2RAGE: magnetization‐prepared 2 rapid gradient echoes sequence
MR or MRI: magnetic resonance imaging
ROI: region of interest
S1: primary sensory cortex
SMA: supplementary motor area
TAF: sound‐paced thumb‐alternating finger opposition
TAFfast: unpaced thumb‐alternating finger opposition
TE: echo time
TIF: sound‐paced thumb‐index finger tapping
TIFfast: unpaced thumb‐index finger tapping
TR: repetition time

## 1 INTRODUCTION

Measuring the neural substrates associated with a motor or cognitive task using functional MRI (fMRI) has been extensively used in neurosciences in the past two decades (Sadraee et al., 2021) and has increasingly been utilized in clinical applications, for example, for preoperative mapping for brain surgery (Jalilianhasanpour et al., 2021; Manan et al., 2021) or neurofeedback therapies (Dudek & Dodell‐Feder, 2021; Thibault et al., 2018). The majority of studies evaluating task‐based brain activation do so by contrasting the blood oxygenation level‐dependent (BOLD) signals during active and control conditions as described by Ogawa et al. (1990). This approach allows for a wide variety of task designs and examination of various neural processes. To be able to draw conclusions and base further research on previous results, the reliability of the measure is utterly critical. However, there are different forms of reliability, and which of its forms is critical depends on the measure and construct under investigation: Internal consistency reliability is crucial for tasks measuring rapidly changing states (e.g. emotions), while interrater (i.e. inter‐scanner) reliability is essential for multicentric studies. Finally, the discovery of traits and prognostic or predictive markers relies on test‐retest reliability.

Reliability of functional imaging has been measured using several different metrics. One of the first and most crude measures to assess reliability is comparing the number of activated voxels. However, this only allows estimating whether the amount of activation is comparable but does not contain information on spatial distribution of this activation. Consequently, this method has fallen out of use for evaluation of fMRI reliability (Cohen & DuBois, 1999). An alternative approach that assesses the spatial distribution of activation by measuring the spatial overlap of brain activation can be performed using the Dice or Jaccard coefficients (Dice, 1945; Jaccard, 1912). This form of reliability is especially crucial for studies aiming to identify brain regions that are involved in a specific task. Finally, intraclass correlation coefficients (ICCs) can additionally assess the amplitudes or weights of activations within voxels or regions of interest (ROIs) over time (Shrout & Fleiss, 1979). Reliable amplitudes are crucial when correlational or regression analyses are planned.

Several publications have reported low reliability of task‐based fMRI, and a recent meta‐analysis by Elliott et al. reported low test–retest reliability across various tasks, especially on the single‐subject level (Bennett & Miller, 2013; Elliott et al., 2020). Poor subject‐level reliability sets limits to the minimal observable effect sizes for correlational analyses. However, examination and comparison of activated regions among groups is still the most frequently used form of analysis and utilizes spatial group‐level reliability of superthreshold clusters of voxels. Moreover, a large proportion of the literature on fMRI task reliability is based on data from older scanners. Considering the tendency to higher field strengths, shorter TR, acquisition acceleration and optimized processing pipelines, more studies assessing reliability in modern settings are needed.

Although the average test–retest reliability in the meta‐analysis of Elliott et al. was poor, there was a large range across the included studies, suggesting that the reliability of task‐based fMRI might vary on the specific task and its implementation. Interestingly, four of the 10 studies with the highest reliability used motor tasks: Friedman et al. (2008) examined paced alternating button pressing with audiovisual cues, Rath et al. (2016) investigated fist‐clenching, Estevez et al. (2014) studied robot‐assisted elbow motion and Kimberley et al. (2008) used a drawing task. Motor tasks may be ideal to examine test–retest reliability, as the targeted brain regions are well characterized. One of the most classic motor tasks is finger tapping. Body movement may induce head movement in the scanner, which should be minimized. Finger‐tapping tasks allow minimization of the coupling of body and head movement, as the hands can usually move freely even in the confined space of a scanner and with elbows fixed for stabilization. Frequent implementations of this task are the use of a button box or free‐moving thumb‐finger opposition. Both button pressing and thumb‐finger opposition fMRI test–retest reliability have been examined in the past (Ibinson et al., 2022; Lee et al., 2010; Marshall et al., 2004; Yoo et al., 2005). However, button pressing might differ from the more naturalistic thumb‐finger opposition, especially in fast, unpaced paradigms, and there are surprisingly few reliability studies of these tasks, considering their frequent use. Moreover, the studies examining thumb‐finger opposition were either conducted with longer repetition time (TR ≥ 3 s) and in lower field strength (i.e., 1.5 T) than the current standard, examined only one variation of finger tapping (either externally or self‐paced), or restricted the analyses to either spatial overlap or ROI activation amplitude comparison. In this study, we aimed to assess spatial and amplitude test–retest reliability of an fMRI task investigating fine motor behaviour on group‐level testing multiple versions of finger tapping in two separate sessions 3 weeks apart. We expected relatively consistent activation of a motor network most pronounced in left primary motor and sensory cortex (M1, S1), premotor cortex, supplementary motor area (SMA), parietal regions, basal ganglia and right cerebellum (Witt et al., 2008).

## 2 MATERIALS AND METHODS

### 2.1 Participants

We recruited 42 right‐handed healthy participants from the general population in Switzerland as a control group for a larger project (OCoPS‐P, ClinicalTrials.gov identifier: NCT03921450). Participants were recruited via advertisements and word of mouth. Inclusion criteria were right‐handedness as confirmed by the Edinburgh Handedness Inventory (Oldfield, 1971), age between 18 and 60 years and ability and willingness to participate in the study. Exclusion criteria were substance abuse other than nicotine, history of psychiatric disorders or medical conditions impairing movements, epilepsy, history of head trauma with loss of consciousness and contraindications for MR scans, that is, metal objects in the body or pregnancy. Written informed consent was obtained from all participants. The study protocol adhered to the Declaration of Helsinki (World Medical Association, 2013) and was approved by the local ethics committee (KEK‐BE 2018‐02164). Out of 42 participants, 31 data sets were included in the analyses. Reasons for exclusion were withdrawal of consent (*n* = 2), cancellation of the second session due to the COVID‐19 pandemic (*n* = 3), technical/language issues (*n* = 2), insufficient task performance with at least one of the task conditions never performed correctly (*n* = 2) and excessive motion in the scanner (*n* = 2). Demographic characteristics and task performance in fast conditions are provided in Table 1.

**TABLE 1:** Sample characteristics and task performance

	Baseline (mean ± SD)	Follow‐up (mean ± SD)
Age (years)	35.7 ± 12.2	
Sex (*n*, % female)	16 (51.6)	
Education (years)	16.2 ± 3.3	
TIFfast performance (Taps/s)	3.71 ± 1.15	3.86 ± .95
TAFfast performance (Taps/s)	3.00 ± .91	3.05 ± .86

Abbreviations: TAF, paced thumb‐alternating finger opposition; TIF, paced thumb‐index finger tapping; TIF/TAFfast, unpaced condition with movement as fast as possible.

### 2.2 Image acquisition

Participants underwent two imaging sessions that were scheduled 3 weeks apart at the same hour of the day. At both sessions, we acquired structural and functional neuroimaging data at the Translational Imaging Center Bern of sitem‐insel Bern on a 3T Magnetom Prisma scanner (Siemens Healthcare, Erlangen, Germany). First, we acquired structural T1‐weighted images (MP2RAGE, 176 slices, FOV 240 × 256 mm, voxelsize 1 × 1 × 1 mm, TR = 5000 ms, TE = 2.98 ms, flip angles = 4°/5°) and then task‐based fMRI (mbep2d, 660 volumes, covering 11 min, 72 slices, FOV 230 × 230 mm, voxelsize 2.5 × 2.5 × 2.5 mm, TR = 1000 ms, TE = 37 ms, flip angle = 30°).

### 2.3 fMRI task

The task was in a block design with five repetitions of four movement conditions, with a fixed duration of 17 s for each block. Active conditions were separated by two different control conditions of random length between 12 and 17 s. The order of active and control conditions remained consistent across all repetitions and sessions. Subjects performed all tasks with the right hand. Participants were instructed verbally before the scans and written cues were displayed via a projector at the beginning of each condition.

**FIGURE 1:**
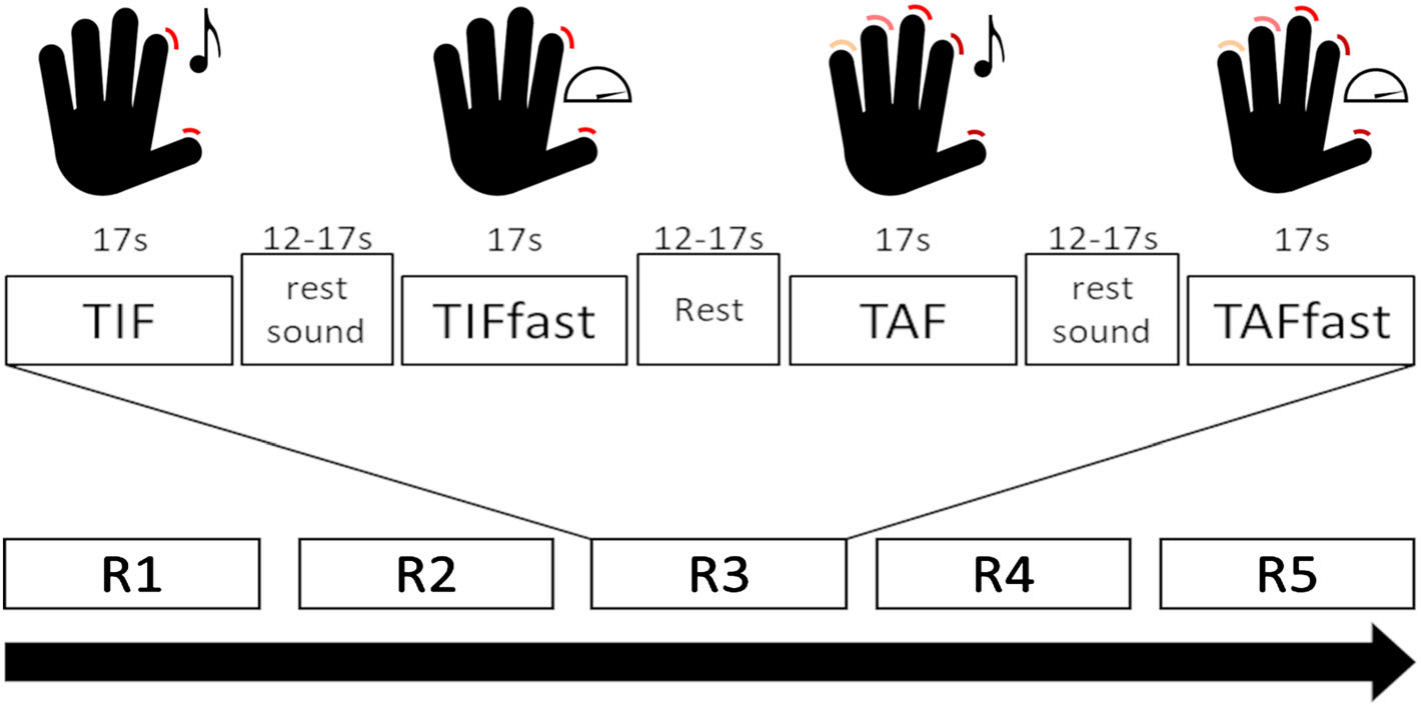
Schematic depiction of the examined finger‐tapping task. TIF, paced thumb‐index finger tapping; TAF, paced thumb‐alternating finger opposition; TIFfast/TAFfast, unpaced condition with movement as fast as possible; R1–R5: runs (repetitions). Mean difference of active conditions and their respective rest condition was used for group analyses.

The four active conditions consisted of (i) sound‐paced thumb‐index finger tapping (TIF) at .5 Hz; (ii) unpaced, as fast as possible thumb‐index finger tapping (TIFfast); (iii) paced thumb‐alternating finger opposition (TAF) at .5 Hz and (iv) unpaced, as fast as possible thumb‐alternating finger opposition (TAFfast). The rest conditions following paced active conditions were combined with the pacing sound and the instruction to listen but not move. Runs were separated by a short break with a length between 6 and 12 s. When no instructions were displayed, a fixation cross was presented in all conditions. See Figure 1 for a schematic depiction of the task design. Stimuli were presented, and onsets of conditions were logged using E‐Prime (Version 2.0.10 Psychology Software Tools, Pittsburgh, PA, USA). Sounds were delivered via MR‐safe headphones. We videotaped participants' hands during the task and verified the correct execution of each condition. Additionally, to evaluate the reliability of motor performance, we counted the number of taps/oppositions for the fast movement conditions.

### 2.4 Preprocessing and first‐level analysis

Preprocessing was performed in SMP12 (Revision 7771, Welcome Trust, London, UK, https://www.fil.ion.ucl.ac.uk/spm/) and MATLAB (R2020b, MathWorks, Natick, USA) and was identical for both sessions. The MP2RAGE sequence acquires images at two inversion times and calculates a unified resulting image with higher cerebral tissue contrast but increased extracerebral noise that may interfere with segmentation and co‐registration (Marques et al., 2010). Therefore, we masked the unified image with the thresholded second inversion image to suppress the background. Then, we applied segmentation and normalization to MNI space within CAT12 (Christian Gaser 2018, http://www.neuro.uni-jena.de/cat/), and smoothing with a 5‐mm full width at half maximum (FWHM) kernel to structural images. Functional images were realigned, co‐registered to the corresponding structural image, normalized using the DARTEL (Ashburner, 2007) approach and smoothed using a 5‐mm FWHM kernel. Subjects with mean framewise displacement >.5 mm or displacement >2.5 mm in one of the three translations or >2.5° in one of the three rotations were excluded from the analysis.

We built subject‐wise first‐level models in SPM12 with one regressor for each of the conditions (four movement conditions and two rest conditions), as well as regressors for each of the three translations and three rotations from realignment as covariates. We then contrasted beta‐values of each of the four movement conditions with the corresponding rest condition (TIF, Listen; TIFfast, Rest; TAF, Listen; TAFfast, Rest), as well as all tapping–all resting conditions. The resulting beta‐difference maps were the input of the group‐level ICC analyses, whereas we used the resulting t‐maps for group‐level overlap analyses.

### 2.5 Statistical analyses

To assess reliability of task performance, we calculated the average number of taps per second for the unpaced (fast) conditions and conducted paired *t* tests and intraclass correlation coefficient (ICC_3,k_) analyses between both sessions for these performance metrics in R (version 4.0.3, The R Foundation for Statistical Computing).

We applied several strategies to evaluate imaging reliability. First, we explored differences in activation amplitude between sessions. Second, we examined the overlap of significant activations across the sessions to evaluate consistency of their spatial distribution. Finally, we calculated ICCs to investigate consistency of activation amplitudes in three sets of ROIs. We modelled paired *t* tests between sessions for each of the four imaging contrasts in SPM to evaluate whether there were significant differences in activations between sessions. To evaluate spatial similarity of activations, we calculated the Dice similarity coefficients (DSCs) for each contrast. The DSC is a simple measure for the overlap of clusters and is defined as

$$\mathrm{DSC}=\frac{2*|X⋂Y|}{\left|X\right|+|Y|},$$

where *X* and *Y* are the extent of each session's activations at a given threshold (Dice, 1945; Rombouts et al., 1997). Since the DSC is highly dependent on the chosen threshold (Duncan et al., 2009; Fernandez et al., 2003), we performed these analyses with three different thresholds: first, *p* = .05 to capture and compare as much activation as possible and then the two standard thresholds of SPM *p* = .001 and family‐wise error corrected *p*_FWE_ = .05 (~*p* = 4.7239e‐07). We did not apply any cluster forming threshold. DSC provides information on spatial reliability of activations and is distinct from *t* tests: DSCs compare extent and localization of significant activations between sessions, depicting spatial similarity of these activations. *T* tests compare amplitudes of all (de)activations, including nonsignificant ones, depicting amplitude differences. It is important to note that incongruences in DSC analyses do not necessarily relate to significant differences in *t* tests.

Additionally, to assess reliability of amplitudes, we extracted contrast values in three different sets of ROIs and calculated intraclass correlation coefficients ICC_(3,k)_ between sessions. ICC_(3,k)_ (hereafter ICC) is

$${\mathrm{ICC}}_{\left(3,k\right)}=\frac{\mathrm{BMS}-\mathrm{EMS}}{\mathrm{BMS}},$$

where BMS is the between‐subject mean square and EMS is the error mean square (Shrout & Fleiss, 1979). Therefore, the ICC depicts the proportion of true variance in the total variance. ICC analysis has become a standard for several types of reliability analyses. DSC allows examination of spatial distribution of two categories (activated and not activated), whereas ICC allows examination of amplitude reliability in an ROI.

ROIs can be the primary unit of analysis and are often defined a priori based on previous literature or anatomical regions. Another frequent use of ROIs is to define them based on significant clusters from a whole‐brain analysis to examine correlations with a variable of interest. To account for these different modes of ROI construction, we examined three sets of ROIs: first, a set of spheres with 10‐mm radius centred on peaks reported in an activation likelihood estimation by Hardwick et al. (2013) (Table S1 and Figure S1). Because of the proximity of bilateral M1 and S1 peaks, they share 40% of their volumes. To ensure consistent ROI creation, we did not modify these ROIs. Second, a set consisting of anatomical ROIs exported from the AAL‐atlas (Tzourio‐Mazoyer et al., 2002) (Table S2 and Figure S2), and finally a functionally defined set for which we conducted a conjunction analysis of activations of both sessions for each tapping condition and defined significant clusters at a threshold of *p*_FWE_ < .05 as ROIs (Table S3 and Figures S3–S7). Note that the three sets differ in shape, size and location of the ROIs despite similar naming (Figure S8).

To assess the influence of the broad range of age on the observed reliability, we split the sample in half, doing a median split at 31 years and repeated all test–retest reliability analyses in both age groups. To our knowledge, there is no consensus regarding the interpretation of DSC. Therefore, we will apply the guidelines of Cicchetti (1994) to both DSC and ICC values. Coefficients below .4 will be considered poor; between .4 and .59, fair; between .6 and .74, good and >.75, excellent.

## 3 RESULTS

### 3.1 Tapping performance and reliability

Tapping performance for the two unpaced (fast) conditions is provided in Table 1. Participants tapped slightly faster in thumb‐index finger tapping than in thumb‐alternating finger opposition (Δ = .76, 95% confidence interval [CI] .40–1.11, *p* < .001). Paired *t* tests of performance showed no significant improvement over time (TIFfast: mean difference = .14 Taps/s, *p* = .12; TAFfast: mean difference = .05 Taps/s, *p* = .58). ICCs indicated excellent reliability of performance in both conditions (TIFfast: ICC = .94, 95% CI .90–.97; TAFfast: ICC = .92, 95% CI .86–.96).

In the paced conditions, the number of trials excluded due to incorrect tapping was comparable for TIF (2.6%) and TAF (3.2%) (*Χ* ^2^ = .1, *p* = .75). For TAF, more trials were excluded at follow‐up (5.8%) than at baseline (.01%) (*Χ* ^2^ = 4.9, *p* = .027). Similarly but statistically only at trend level, more TIF trials were excluded at follow‐up (4.5%) than at baseline (.01%) (*Χ* ^2^ = 3.13, *p* = .08).

### 3.2 Activations

All contrasts showed the expected activations in the motor network in response to right‐hand finger tapping: left primary motor and sensory cortices, premotor and SMAs and bilateral cerebellum. Additionally, all contrasts but TIF showed activations in left parietal and bilateral frontal cortices, as well as in subcortical structures, such as putamen or thalamus (see Table S3 for clusters in conjunction analyses of each contrast).

### 3.3 Imaging reliability

Paired *t* tests of fMRI task activations showed no significant differences between sessions for any of the five contrasts at *p*_FWE_ < .05. However, at *p* < .001 and *p* < .05, we found clusters with higher activation at follow‐up in bilateral precuneus for TIFfast, TAFfast and all tapping vs. all rest. Additionally, three clusters in bilateral operculum and left cerebellum showed higher activation at baseline than at follow‐up for TIF at *p* < .05.

**FIGURE 2:**
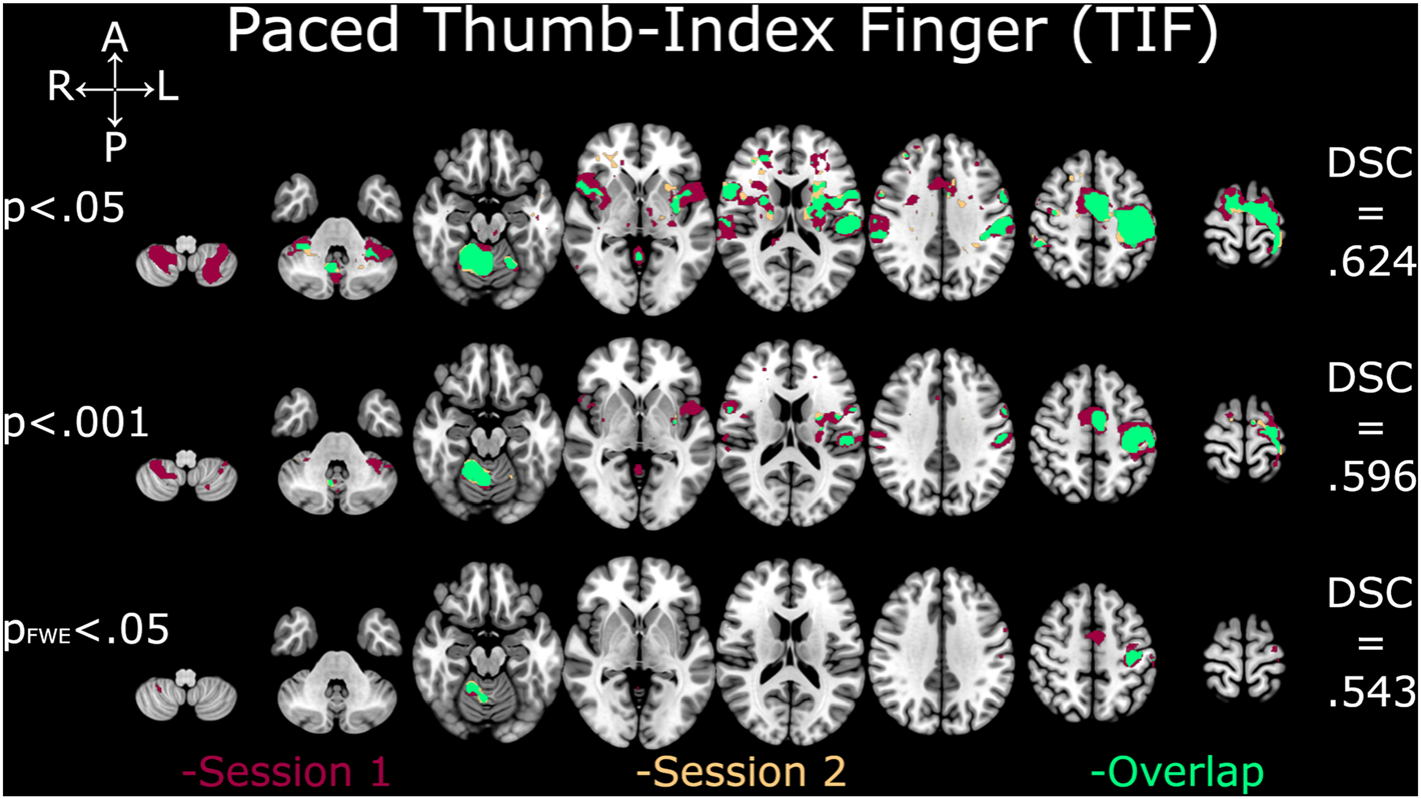
Depiction of Dice similarity coefficients for paced thumb‐index finger tapping (TIF). Single sessions and overlapping activation of TIF at the three examined thresholds. DSC, Dice similarity coefficient; FWE, family‐wise error

**FIGURE 3:**
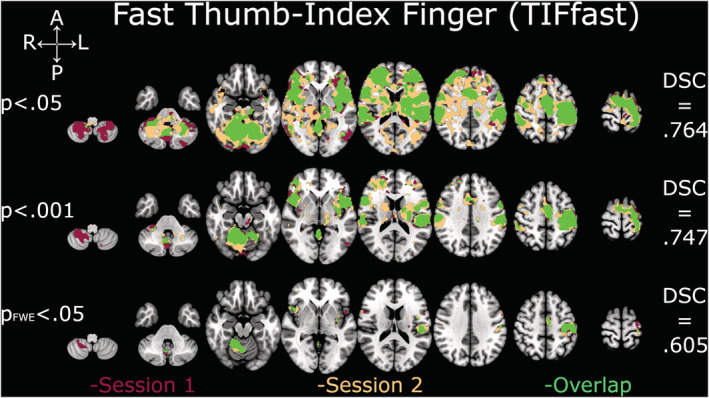
Depiction of Dice similarity coefficients for unpaced thumb‐index finger tapping (TIFfast). Single sessions and overlapping activation of TIFfast at the three examined thresholds. DSC, Dice similarity coefficient; FWE, family‐wise error

**FIGURE 4:**
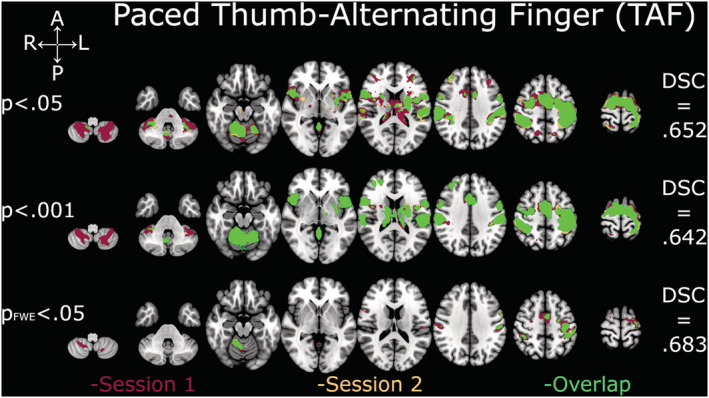
Depiction of Dice similarity coefficients for paced thumb‐alternating finger opposition (TAF). Single sessions and overlapping activation of TAF at the three examined thresholds. DSC, Dice similarity coefficient; FWE, family‐wise error

**FIGURE 5:**
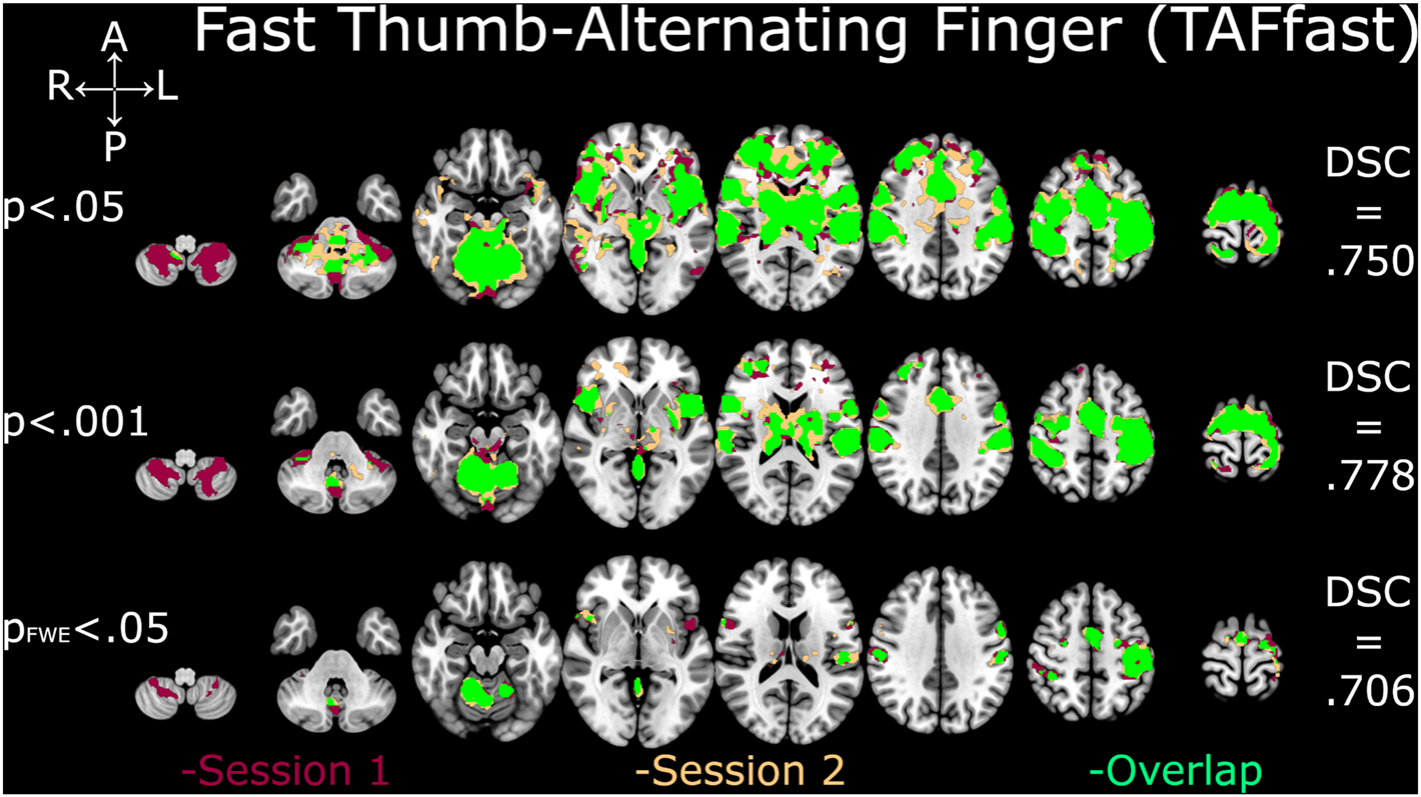
Depiction of Dice similarity coefficients for unpaced thumb‐alternating finger opposition (TAFfast). Single sessions and overlapping activation of TAFfast at the three examined thresholds. DSC, Dice similarity coefficient; FWE, family‐wise error

**FIGURE 6:**
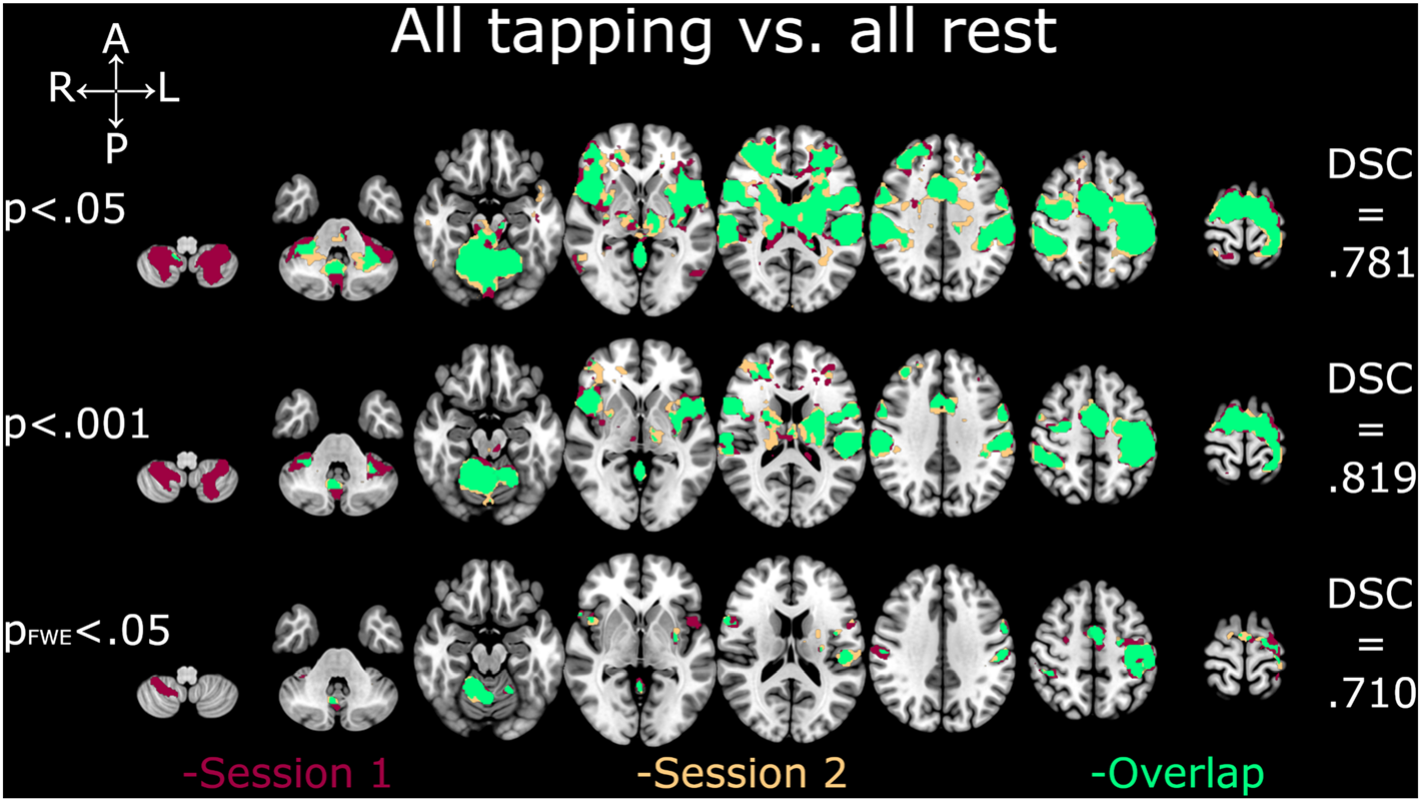
Depiction of Dice similarity coefficients for all‐tapping conditions vs. all‐rest conditions. Single session and overlapping activations of all‐tapping vs. all‐rest conditions at the three examined thresholds. DSC, Dice similarity coefficient; FWE, family‐wise error

DSC analyses yielded comparable coefficients across all thresholds and contrasts (Figures 2, 3, 4, 5, 6). The overlap between the two sessions was good to excellent in all cases, except for TIF at the two lower thresholds (.001 and FWE‐.05; Figure 2), which were in the fair range. The all‐tapping vs. all‐rest contrast yielded the highest DSC values for all three thresholds (Figure 6). The DSC values are provided in Table 2.

**TABLE 2:** Dice similarity coefficients (DSC)

Contrast	TIF	TIFfast	TAF	TAFfast	AllC
Threshold *p* = .05	.624	.764	.652	.750	.781
Threshold *p* = .001	.569	.747	.642	.778	.819
Threshold *p*_FWE_ = .05	.543	.605	.683	.706	.710

Abbreviations: FWE, family‐wise error rate; TAF, paced thumb‐alternating finger opposition; TIF, paced thumb‐index finger tapping; TIF/TAFfast, unpaced condition with movement as fast as possible.

ICCs in literature based, 10‐mm spherical ROIs showed a large range and variability (.04–.91). The ROIs with at least fair ICC for all contrasts were bilateral primary motor and sensory cortices, SMA and left putamen (Table S1). There were no ROIs with poor ICC for all contrasts, but left cerebellum and bilateral thalamus had no good or excellent ICC in any of the contrasts.

The atlas‐based ROIs showed a relatively large range of ICCs (.08–.81). ROIs with at least fair reliability in all contrasts were bilateral primary sensory cortex and cerebellum, left putamen and right SMA. Bilateral primary motor cortices showed poor ICCs only for the paced thumb‐alternating finger contrast (Table S2). Again, no ROI had poor reliability in all contrasts, but bilateral thalamus, left SPL and right putamen had no good or excellent ICC in any of the conditions.

Finally, ICC in ROIs based on conjunction analyses showed a similar variability with most values in fair, good and excellent ranges. Range of ROI size was immense with seven to 4451 voxels, as was range of ICC with .13–.74. The all‐tapping vs. all‐rest contrast was the only one with at least fair ICCs in all ROIs (Table S3).

The average ICCs were in the fair range for paced and unpaced TIF and TAF contrasts for all three ROI sets, except TIFfast (good) and TAF (poor) in the atlas‐based ROIs, whereas the all‐tapping vs. all‐rest contrast yielded average ICCs in the good range in all three ROI sets (Table 3).

**TABLE 3:** Average intraclass correlation coefficients (ICCs) per contrast and region of interest (ROI) set

	TIF	TIFfast	TAF	TAFfast	AllC	Average across contrasts
Literature	.48 ± .15	.54 ± .21	.45 ± .18	.54 ± .19	.61 ± .14	.52 ± .06
Atlas	.53 ± .08	.62 ± .10	.33 ± .17	.52 ± .14	.63 ± .11	.53 ± .12
Conjunction	.48 ± .18	.54 ± .18	.44 ± .14	.49 ± .16	.60 ± .10	.51 ± .06

*Note*: Mean ± SD of ICCs per tapping contrast and ROI set.

Abbreviations: TAF, paced thumb‐alternating finger opposition; TIF, paced thumb‐index finger tapping; TIF/TAFfast, unpaced condition with movement as fast as possible.

### 3.4 Age groups

Characteristics and tapping performance of age groups are provided in Table S4. They did not differ in sex (*Χ* ^2^ = .8; *p* = .37), education (*t* = .97; *p* = .34) or tapping performance (all *p* > .55). Both groups tapped slightly faster in thumb‐index finger tapping than in thumb‐alternating finger opposition (Δyoung = .82; *p* < .001, Δold = .69; *p* < .001), but paired *t* tests of performance showed no significant difference between the sessions in the younger or older half of the sample (all *p* > .18). ICCs indicated excellent reliability of performance in both conditions in both groups (all ICC ≥ .92).

For the two more liberal thresholds (*p* < .05 and *p* < .001), overlap was in the fair‐to‐good range in both groups for all conditions but TIF. The younger group had poor overlap for this contrast at all thresholds. The older half of our sample showed numerically higher overlap in all cases but the most liberal threshold (*p* < .05) in the all‐tapping vs. all‐rest condition. We noticed a sharp drop in overlaps between *p* < .001 and *p*_FWE_ < .05 in all conditions for both age groups. Comparison of all DSC between the younger and older half of the sample using a Mann–Whitney *U*‐test showed no significant difference. Since there was a sharp drop of coefficients at *p*_FWE_ < .05, we also compared the DSC for the two more liberal thresholds between the age groups and again found no significant difference. Dice coefficients per age group and condition are provided in Table S5 and Figure S8.

Separate ICC analyses in the age groups showed averages of ICCs in the fair‐to‐good range for most conditions using the literature‐based or conjunction‐based ROIs, regardless of age group (Table S6). The anatomical, atlas‐based ROIs had averages of ICCs in the poor range for three conditions in the younger half of the sample. Again, in most direct comparisons, the older half of the sample showed numerically higher reliability than the younger half. Additionally, we compared the ICCs per age group, condition and ROI category using two‐sample *t* tests. In nine out of 18 comparisons, ICCs were significantly higher in the older half of the sample, whereas the younger half had higher ICCs in only one comparison. There was no significant difference in the remaining eight comparisons (see Table S6).

## 4 DISCUSSION

In the present study, we evaluated the test–retest reliability of fMRI‐derived brain activations for four simple motor tasks in a right‐handed healthy population. We found good reliability regarding spatial distribution and satisfactory reliability for amplitudes of activations on group level.

Regarding task performance, participants showed no significant improvement across the two sessions in both unpaced movement conditions. Therefore, we may assume that there was no relevant training effect. This is in line with literature; although within‐ and between‐session training effects have been shown for an intersession interval of 24 h, no training effect was observed at an interval of 2 weeks (Nguemeni et al., 2021; Sardroodian et al., 2016). Reliability of tapping performance of both unpaced conditions was excellent with ICCs of >.9, demonstrating behavioural robustness of the motor tasks themselves. The increased number of trials with incorrect paced tapping might hint at a reduction in motivation or attention at follow‐up compared with baseline.

As expected, we detected activations in left primary motor and sensory cortex, left premotor cortex, left SMA and right cerebellum for all tasks. Again, this is in line with literature (see Witt et al., 2008, for an ALE meta‐analysis). In the relatively more demanding conditions, additional brain regions were recruited, that is, alternating finger opposition evoked activity in more regions than index finger tapping and unpaced, fast tapping recruited more regions than paced, slower tapping. Furthermore, clusters of activated voxels tended to be larger in conditions that are more demanding, reflecting the increased need of neural resources for these task conditions (Goble et al., 2010; Van Impe et al., 2013). The higher signal at follow‐up in precuneus during the fast conditions might actually represent a weaker deactivation of the default mode network that is associated with mind wandering (Buckner et al., 2008; Fox et al., 2015), possibly reflecting a reduction of focus at follow‐up.

We found good‐to‐excellent spatial activation overlap in all five contrasts with little variance across all three tested statistical thresholds for activation maps (*p* < .05; *p* < .001; *p*_FWE_ < .05), as demonstrated by the DSCs. This demonstrates reliable spatial identification of activated voxels in response to finger tapping at the most commonly applied thresholds. The all‐tapping vs. all‐rest contrast had the largest overlap at all three thresholds, but the differences were relatively small. This indicates that fMRI can reliably identify the brain regions activated in response to these tasks at group level. Good test–retest spatial overlap has been reported for several task designs, but the range of reported overlaps was immense even among finger‐tapping tasks (Bennett & Miller, 2010; Gountouna et al., 2010; Ibinson et al., 2022; Yetkin et al., 1996). The larger overlap of the fast, unpaced compared with the paced contrasts might reflect the behavioural performance: Most of the volume of non‐overlap for the paced contrasts consisted of activations at baseline that were missing at follow‐up, possibly paralleling the higher number of correct runs at baseline for these contrasts. Conversely, the non‐overlap for the fast contrasts included more activation only during follow‐up. We found no significant difference in tapping performance in the fast conditions, but there was a subtle numerical increase of tapping speed at follow‐up. Moreover, the larger volume of activation in the more demanding fast conditions might represent recruitment of a higher proportion of available neural resources, resulting in a smaller volume for potential non‐overlap.

Similarly, ROI‐based analyses of ICC showed a large span of reliability of activation amplitude across ROIs. Interestingly, we found ICCs of >.4 in most ROIs independent of the mode of ROI selection. This was unexpected, as the sets of ROIs differed in shape, size and location of the ROIs with potentially little overlap between them (see Figure S8). However, average ICC per contrast was only in the fair range in conjunction‐ and literature‐based ROIs. In the anatomically defined ROIs, TAF had poor average ICC, whereas TIFfast was in the good range. Notably, the all‐tapping vs. all‐rest contrast showed the least amount of variability with all but one ROIs having at least fair reliability and average ICCs being in the good range for all three methods of ROI definition. These results indicate that it is possible to associate amplitudes of activations with other variables. Similar to the reports on spatial overlap, reported studies on activation amplitudes using test–retest ICC show large variability (Aron et al., 2006; Bennett & Miller, 2010; Friedman et al., 2008). Generally, motor tasks tend to yield higher reliability than cognitive tasks (Bennett & Miller, 2010; Fliessbach et al., 2010). However, Havel et al. (2006) reported hand movements to have higher reliability than movement in other anatomical regions, pointing to differences even between motor tasks. Regarding the variability within the ROI analyses, S1 and M1 bilaterally seem to have superior ICCs across most contrasts, whereas there is no detectable pattern in the other regions. We suggest that M1 and S1 are consistently recruited during the finger‐tapping tasks and therefore achieve higher ICCs than areas with different functional specialization. It remains to be established whether studies in much larger samples would detect interpretable patterns of ICC distribution.

In both the spatial overlap and ICC analyses, the all‐tapping vs. all‐rest contrast had the highest reliability. However, this is also the least specific contrast. In the present case, the higher number of trials and the longer acquisition period included in the more general contrast may have led to statistically more robust but less specific responses (Gordon et al., 2017). Moreover, outlier responses to specific tasks loose impact through averaging across several subjects and trials. Extending this notion in the opposite direction, this may explain the low subject‐wise reliability that recent studies reported (Elliott et al., 2020), as the total number of trials in a single subject is usually substantially lower than the number of trials in a whole group of subjects. Friedman et al. (2008) previously demonstrated this relationship of number of trials and reliability on the group level in a finger‐tapping task. It is important to note that we aimed to examine group‐level reliability in the present study. This is reflected by the design of our task that allows for a maximum of five trials per tapping condition. Moreover, high group‐level reliability does not necessitate high subject‐level reliability and vice versa (Frohner et al., 2019; Gordon et al., 2017). However, examination of single subject test–retest reliability may inform the interpretation of group‐level reliability. For example, high group reliability with low subject reliability would argue for a population tendency of a state that is unstable in the individual, whereas low group but high individual reliability could reflect heterogeneity in stable individual traits.

Various factors other than acquisition duration and number of trials have been reported to increase reliability of task‐based fMRI: Shorter between‐session interval, block‐design had higher reliability than event‐related, cortical activations had higher reliability than subcortical ones, healthy populations generated more robust results than patients, but evidence on the effect of these factors is conflicting (Bennett & Miller, 2010, 2013; Elliott et al., 2020). Moreover, we found numerically higher reliability in the older half of the study sample compared with the younger half for both overlap and amplitudes of activations. However, this did not pertain to the motor behaviour itself. Larger between‐subject variability may have increased ICCs in the older half and may have had a smoothing effect in the overlap analyses.

For research contexts, reliability should not be examined in isolation, as larger effect sizes as well as larger sample sizes can enhance the detection of effects even with less reliable measures. Moreover, there are sources of uncertainty beside the reliability of the BOLD signal in fMRI: Even when evaluating the same set of images, there is substantial variability depending on the choice of toolbox for the analyses, the preprocessing, models and even operating systems, computers and versions of the toolboxes (Bowring et al., 2019; Carp, 2012; Pauli et al., 2016).

Some limitations require consideration for this study. First, our sample size is limited. However, it is in the range of typical fMRI studies. The limited sample size prevented more in‐depth investigation of possible age effects, and our median split resulted in age groups with vastly different age ranges, as the younger half spanned 12 years, whereas the older half spanned 24 years. Second, we examined reliability between only two sessions. Reliability between multiple sessions might differ from the one observed here. Third, we had a limited number of trials per condition, as the task consisted of five runs and some trials were excluded because of incorrect tapping. Moreover, since our sessions took place 3 weeks apart, the female participants in the present sample were probably in different phases of their menstrual cycle in the two sessions. Effects of the menstrual cycle on brain networks, including the somatomotor network, and motor behaviour have been demonstrated (Bayer & Hausmann, 2012; Pellegrini et al., 2018; Pritschet et al., 2020). Finally, there is an unknown amount of true variability that is unrelated to the measure. The true neural response to the task may vary because of subject‐ and session‐specific variables, such as participants being tired or varying concentration and motivation. In fact, the increased number of incorrect paced trials and the higher signal in precuneus during unpaced trials at follow‐up are suggestive of differences in focus between the sessions.

## 5 CONCLUSION

In sum, the presented tapping tasks can reliably identify brain regions that are activated in response to the task. Test–retest reliability was good in spatial and fair in amplitude domain on group level. Subject‐ and group‐level reliability are distinct properties of a task, and task design should reflect the level of intended analyses (i.e., subject vs. group). Although the reliability of the amplitudes was often only in the fair range and caution is warranted when examining correlations of activations with other variables, identification of activated regions in response to a task and their comparisons between groups are still a valid and important mode of analysis in neuroimaging to find population tendencies and differences.

## AUTHOR CONTRIBUTIONS

**Florian Wüthrich:** Data curation; formal analysis; investigation; methodology; visualization; writing‐original draft; writing‐review and editing. **Stephanie Lefebvre:** Conceptualization; data curation; investigation; methodology; supervision; validation; writing‐review and editing. **Niluja Nadesalingam:** Data curation; validation; writing‐review and editing. **Jessica Bernard:** Investigation; methodology; writing‐review and editing. **Vijay A Mittal:** Funding acquisition; investigation; writing‐review and editing. **Stewart A Shankman:** Funding acquisition; investigation; writing‐review and editing. **Sebastian Walther:** Funding acquisition; investigation; supervision; validation; writing‐original draft; writing‐review and editing.

## ETHICS STATEMENT

Written informed consent was obtained from all participants. The study protocol adhered to the Declaration of Helsinki and was approved by the local ethics committee.

## CONFLICTS OF INTEREST

SW has received honoraria from Janssen, Lundbeck, Mepha, Neurolite and Sunovion. All other authors report no conflicts of interest.

### PEER REVIEW

The peer review history for this article is available at https://publons.com/publon/10.1111/ejn.15865.

## Supporting information


**Table S1.** ‐ Intraclass Correlation Coefficients in spherical ROIs with r = 10 mm based on ALE[1].
**Table S2.** ‐ Intraclass Correlation Coefficients in anatomical ROIs based on aal‐atlas[2].
**Table S3.** ‐ Intraclass Correlation Coefficients in ROIs based on conjunction analyses at threshold pFWE < .05.
**Table S4.** Sample characteristics and performance.
**Table S5.** ‐ Dice Similarity Coefficients (DSC).
**Table S6.** – Average ICC values per condition and age group.
**Figure S1.** Depiction of the literature‐based ROI‐set. ROIs were created by drawing spheres with 10 mm radius centered on peaks reported in an activation likelihood estimation[1]. Note the overlap of bilateral M1 and S1 ROIs (40% overlap).
**Figure S2.** Depiction of the anatomical ROI‐set from the AAL‐atlas[2].
**Figure S3.** Depiction of ROIs derived from conjunction analysis of paced Thumb – Index Finger tapping vs. Listen contrast (TIF) at threshold pFWE < .05.
**Figure S4.** Depiction of ROIs derived from conjunction analysis of unpaced Thumb – Index Finger tapping vs. Rest contrast (TIFfast) at threshold pFWE < .05.
**Figure S5.** Depiction of ROIs derived from conjunction analysis of paced Thumb – Alternating Finger opposition vs. Listen contrast (TAF) at threshold pFWE < .05.
**Figure S6.** Depiction of ROIs derived from conjunction analysis of unpaced Thumb – Alternating Finger opposition vs. Rest contrast (TAFfast) at threshold pFWE < .05.
**Figure S7.** Depiction of ROIs derived from conjunction analysis of all tapping conditions vs. all rest conditions contrast (AllC) at threshold pFWE < .05.
**Figure S8.** Overlay of ROI‐sets. Red: Anatomical atlas ROIs; Yellow: Literature‐based ROIs; Green: Example of conjunction analysis based ROIs (AllC) at threshold pFWE < .05.
**Figure S9.** Side‐by‐side comparison of activation overlap by age group and contrast at threshold of p < .001. TIF: Paced thumb‐index finger tapping; TAF: paced thumb alternating finger opposition; TIF/TAFfast: unpaced condition with movement as fast as possible; All: Contrast of all tapping conditions vs. all rest conditions; young: younger hal1f of the study sample (n = 16); old: older half of the study sample (n = 15); DSC: Dice similarity coefficient.

## Data Availability

The data used in this study are available from SW upon reasonable request.
