# Supplementary material for: Test–retest reliability of a finger‐tapping fMRI task in a healthy population
Source: Eur J Neurosci. 2022 Nov 25;57(1):78–90. doi: 10.1111/ejn.15865 (PMC9990175; doi:10.1111/ejn.15865)
Supplement: Supplementary file 1 — Table S1. ‐ Intraclass Correlation Coefficients in spherical ROIs with r = 10 mm based on ALE[1]. Table S2. ‐ Intraclass Correlation Coefficients in anatomical ROIs based on aal‐atlas[2]. Table S3. ‐ Intraclass Correlation Coefficients in ROIs based on conjunction analyses at threshold pFWE < .05. Table S4. Sample characteristics and performance. Table S5. ‐ Dice Similarity Coefficients (DSC). Table S6. – Average ICC values per condition and age group. Figure S1. Depiction of the literature‐based ROI‐set. ROIs were created by drawing spheres with 10 mm radius centered on peaks reported in an activation likelihood estimation[1]. Note the overlap of bilateral M1 and S1 ROIs (40% overlap). Figure S2. Depiction of the anatomical ROI‐set from the AAL‐atlas[2]. Figure S3. Depiction of ROIs derived from conjunction analysis of paced Thumb – Index Finger tapping vs. Listen contrast (TIF) at threshold pFWE < .05. Figure S4. Depiction of ROIs derived from conjunction analysis of unpaced Thumb – Index Finger tapping vs. Rest contrast (TIFfast) at threshold pFWE < .05. Figure S5. Depiction of ROIs derived from conjunction analysis of paced Thumb – Alternating Finger opposition vs. Listen contrast (TAF) at threshold pFWE < .05. Figure S6. Depiction of ROIs derived from conjunction analysis of unpaced Thumb – Alternating Finger opposition vs. Rest contrast (TAFfast) at threshold pFWE < .05. Figure S7. Depiction of ROIs derived from conjunction analysis of all tapping conditions vs. all rest conditions contrast (AllC) at threshold pFWE < .05. Figure S8. Overlay of ROI‐sets. Red: Anatomical atlas ROIs; Yellow: Literature‐based ROIs; Green: Example of conjunction analysis based ROIs (AllC) at threshold pFWE < .05. Figure S9. Side‐by‐side comparison of activation overlap by age group and contrast at threshold of p < .001. TIF: Paced thumb‐index finger tapping; TAF: paced thumb alternating finger opposition; TIF/TAFfast: unpaced condition with movement as fast as possible; All: Contra [file EJN-57-78-s001.pdf]

Supplementary material to:

Test-retest reliability of a finger-tapping fMRI task in a healthy population

Florian Wüthrich[1-3] , Stephanie Lefebvre[1,2], Niluja Nadesalingam[1-3], Jessica A. Bernard[4], Vijay A. Mittal[5-9], Stewart A. Shankman[5,6], Sebastian Walther[1,2]

[1] Translational Research Center, University Hospital of Psychiatry and Psychotherapy, University of Bern, Switzerland

[2]Translational Imaging Center (TIC), Swiss Institute for Translational and Entrepreneurial Medicine, Bern, Switzerland

[3]Graduate School for Health Sciences, University of Bern, Switzerland

[4]Texas A&M University, Department of Psychological and Brain Sciences, Texas A&M Institute for Neuroscience, Texas A&M University, College Station, TX, USA

[5]Northwestern University, Department of Psychiatry and Behavioral Sciences, Chicago, IL, USA.

[6]Northwestern University, Department of Psychology, Evanston, IL, USA

[7]Northwestern University, Institute for Innovations in Developmental Sciences, Evanston/Chicago, IL, USA

[8]Northwestern University, Institute for Policy Research, Evanston, IL, USA

[9]Northwestern University, Medical Social Sciences, Chicago, IL, USA

Test-retest reliability in literature based regions of interest

Table S1 - Intraclass Correlation Coefficients in spherical ROIs with r = 10mm based on ALE[1]						
x y z	Region	ICC TIF (95%-CI)	ICC TIFfast (95%-CI)	ICC TAF (95%-CI)	ICC TAFfast (95%-CI)	ICC AllC (95%-CI)
-26 -54 -26	Left cerebellum	.47 (.02, .71)	.39 (-.13, .67)	.44 (-.04, .69)	.52 (.11, .74)	.52 (.12, .74)
-38 -24 56	Left M1	.72 (.49, .85)	.59 (.24, .78)	.53 (.13, .74)	.53 (.13, .74)	.58 (.22, .77)
-26 4 60	Left PMd	.65 (.35, .81)	.19 (-.49, .56)	.55 (.16, .75)	.46 (.00, .70)	.46 (.02, .71)
-26 4 2	Left putamen	.42 (-.07, .69)	.57 (.21, .77)	.54 (.15, .75)	.67 (.39, .82)	.62 (.30, .79)
-32 -24 62	Left S1	.71 (.47, .84)	.71 (.46, .84)	.70 (.44, .84)	.78 (.59, .88)	.74 (.53, .86)
-30 -56 64	Left SPL	.59 (.25, .78)	.22 (-.43, .58)	.57 (.21, .77)	.37 (-.17, .66)	.65 (.36, .81)
-12 -20 10	Left Thalamus	.42 (-.07, .68)	.40 (-.10, .67)	.04 (-.77, .48)	.41 (-.09, .68)	.33 (-.23, .64)
0 -2 54	Bilateral SMA	.44 (-.02, .70)	.69 (.43, .83)	.44 (-.04, .69)	.44 (-.04, .69)	.51 (.09, .73)
26 -54 -26	Right cerebellum	.31 (-.28, .62)	.56 (.19, .76)	.43 (-.05, .69)	.69 (.43, .83)	.62 (.30, .79)
38 -24 56	Right M1	.57 (.22, .77)	.91 (.83, .95)	.68 (.42, .83)	.80 (.62, .89)	.85 (.72, .92)
26 4 60	Right PMd	.48 (.04, .72)	.41 (-.09, .68)	.21 (-.46, .57)	.58 (.23, .77)	.64 (.34, .81)
26 4 2	Right putamen	.32 (-.26, .63)	.54 (.16, .75)	.43 (-.05, .69)	.43 (-.04, .69)	.60 (.27, .78)
32 -24 62	Right S1	.52 (.11, .74)	.88 (.78, .93)	.46 (.02, .71)	.84 (.70, .91)	.84 (.71, .92)
30 -56 64	Right SPL	.35 (-.20, .64)	.49 (.06, .72)	.51 (.11, .74)	.30 (-.28, .62)	.65 (.36, .81)
12 -20 10	Right Thalamus	.24 (-.40, .59)	.50 (.08, .73)	.19 (-.48, .56)	.20 (-.47, .57)	.47 (.02, .71)

ROI: region of interest; ALE: activation likelihood estimation; ICC: intraclass correlation coefficient; TIF: paced thumb-index finger tapping; TAF: paced thumb alternating finger opposition; TIF/TAFfast: unpaced condition with movement as fast as possible;
M1: primary motor cortex; PMd: dorsal premotor cortex; S1: primary sensory cortex; SPL: superior parietal lobule; SMA: supplementary motor area. M1 and S1 ROIs share 40% of their volume.
Color-coding for ICC-values: red: 0 - .39 (poor); yellow: .40 - .59 (fair); light green: .60 - .75(good); dark green: >.75 (excellent)

Test-retest reliability in anatomical atlas based regions of interest

Table S2 - Intraclass Correlation Coefficients in anatomical ROIs based on aal-atlas[2]

Region	ICC TIF (95%-CI)	ICC TIFfast (95%-CI)	ICC TAF (95%-CI)	ICC TAFfast (95%-CI)	ICC AllC (95%-CI)
Left cerebellum IV-VI	.55 (.18, .76)	.66 (.37, .81)	.45 (-.01, .70)	.62 (.31, .80)	.72 (.48, .85)
Left M1	.64 (.34, .81)	.52 (.12, .74)	.24 (-.40, .59)	.57 (.20, .76)	.48 (.05, .72)
Left putamen	.44 (-.02, .70)	.58 (.22, .77)	.73 (.51, .85)	.66 (.38, .82)	.64 (.34, .81)
Left S1	.60 (.26, .78)	.67 (.40, .82)	.45 (-.01, .70)	.57 (.21, .77)	.66 (.37, .82)
Left SMA	.48 (.05, .72)	.66 (.38, .82)	.22 (-.44, .58)	.45 (-.01, .70)	.68 (.41, .83)
Left SPL	.59 (.24, .78)	.47 (.02, .71)	.39 (-.13, .67)	.31 (-.27, .63)	.46 (.00, .71)
Left thalamus	.38 (-.14, .66)	.54 (.15, .75)	.09 (-.68, .50)	.56 (.18, .76)	.53 (.14, .75)
Right cerebellum IV-VI	.69 (.43, .83)	.60 (.27, .78)	.40 (-.10, .68)	.60 (.26, .78)	.73 (.50, .85)
Right M1	.52 (.12, .74)	.77 (.57, .87)	.08 (-.70, .50)	.72 (.48, .85)	.77 (.57, .87)
Right putamen	.46 (.00, .71)	.53 (.14, .75)	.31 (-.26, .63)	.38 (-.14, .66)	.58 (.23, .77)
Right S1	.47 (.02, .71)	.81 (.65, .90)	.41 (-.08, .68)	.60 (.27, .78)	.76 (.57, .87)
Right SMA	.55 (.17, .76)	.74 (.53, .86)	.43 (-.05, .69)	.57 (.21, .77)	.78 (.60, .88)
Right SPL	.48 (.04, .72)	.60 (.26, .78)	.30 (-.28, .62)	.40 (-.10, .68)	.59 (.24, .77)
Right thalamus	.51 (.10, .74)	.55 (.18, .76)	.17 (-.52, .55)	.23 (-.43, .58)	.50 (.09, .73)

ROI: region of interest; ICC: intraclass correlation coefficient; TIF: paced thumb-index finger tapping; TAF: paced thumb alternating finger opposition; TIF/TAFfast: unpaced condition with movement as fast as possible;
M1: primary motor cortex; S1: primary sensory cortex; SMA: supplementary motor area; SPL: superior parietal lobule.
Color-coding for ICC-values: red: 0 - .39 (poor); yellow: .40 - .59 (fair); light green: .60 - .75(good); dark green: >.75 (excellent)

Test-retest reliability in regions of interest based on conjunction analysis of activations

Table S3 - Intraclass Correlation Coefficients in ROIs based on conjunction analyses at threshold $p_{FWE} < .05$

TIF

x y z (n voxels)	Regions	ICC (95%-CI)
-35 -24 53 (626)	Left M1, premotor cortex, S1	.67 (.39, .82)
-3 -4 55 (109)	Bilateral SMA	.50 (.08, .73)
20 -48 -22 (480)	Right cerebellum	.54 (.16, .75)
-29 -50 -30 (31)	Left cerebellum	.23 (-.42, .58)
-49 -24 19 (15)	Left SII	.29 (-.31, .61)
-45 1 9 (10)	Left anterior insula	.64 (.33, .80)

TIFfast

x y z (n voxel)	Regions	ICC (95%-CI)
-35 -24 55 (2019)	Left M1, premotor cortex, SI, SII	.55 (.18, .76)
24 -50 -26 (1342)	Right cerebellum IV-VI, vermis	.55 (.17, .76)
44 11 1 (484)	Right IFG pars opercularis, anterior Insula	.64 (.34, .81)

TAFfast

x y z (n voxel)	Regions	ICC (95%-CI)
-35 -24 53 (4451)	Left M1, S1, SII, SPL, IPL, bilateral SMA, premotor cortex	.35 (-.21, .64)
20 -50 -22 (2355)	Bilateral cerebellum IV – VI, left VIII, IX, Fastigii ncl., interposed Ncl.	.66 (.37, .81)
58 -18 29 (1156)	Right M1, SI, SII, SMG, IPL	.54 (.16, .75)
-57 1 35 (777)	Left IFG pars opercularis, anterior Insula, premotor cortex	.55 (.18, .76)
44 3 5 (671)	Right IFG pars opercularis, anterior Insula	.52 (.12, .74)
40 -12 61 (360)	Right premotor cortex	.64 (.33, .80)
18 -20 21 (46)	Right thalamus	.40 (-.10, .67)
-27 -12 9 (33)	Left putamen	.29 (-.30, .62)

-45 1 7 (388)	Left IFG pars opercularis, anterior Insula	.70 (.45, .84)
-7 -12 51 (228)	Left SMA	.68 (.42, .83)
54 -24 27 (161)	Right rolandic operculum, SMG	.71 (.46, .84)
-59 7 29 (101)	Left IFG pars opercularis	.38 (-.14, .66)
-29 -52 -26 (73)	Left cerebellum VI	.32 (-.26, .63)
-31 -8 -2 (48)	Left putamen	.18 (-.52, .55)
4 -2 67 (17)	Right SMA	.71 (.46, .84)
-7 5 41 (16)	Left MCC	.41 (-.09, .68)
-15 -20 7 (13)	Left thalamus	.65 (.36, .81)
TAF		
x y z (n voxels)	Regions	ICC (95%-CI)
-35 -24 53 (2412)	Left M1, premotor, S1, IPL	.46 (.00, .71)
20 -48 -22 (696)	Right cerebellum IV – VI, vermis	.47 (.02, .71)
40 -38 53 (381)	Right S1	.62 (.30, .79)
-3 -2 53 (341)	Bilateral SMA	.50 (.08, .73)
-27 -54 -24 (197)	Left cerebellum V, VI	.42 (-.06, .69)
-59 7 29 (65)	Left IFG pars opercularis	.31 (-.27, .63)
52 -20 35 (47)	Right S1, IPL	.53 (.13, .74)
30 -6 61 (44)	Right premotor cortex	.13 (-.59, .53)
-41 -2 13 (43)	Left anterior Insula	.56 (.19, .76)
58 9 23 (38)	Right IFG pars opercularis	.31 (-.28, .62)
-25 -2 13 (21)	Left putamen	.58 (.22, .77)
-17 -10 21 (11)	Left thalamus	.44 (-.04, .69)
-15 -20 5 (10)	Left thalamus	.61 (.28, .79)
-31 -10 1 (7)	Left putamen	.21 (-.46, .57)
2 17 35 (7)	Right MCC	.70 (.45, .84)
AllC		
-35 -24 53 (4293)	Left M1, premotor cortex, S1, SII, IPL, bilateral SMA	.45 (-.01, .70)
22 -48 -22 (2074)	Bilateral cerebellum IV – V, vermis	.57 (.20, .76)
58 -18 29 (796)	Right SMG, S1, SII, IPL	.67 (.39, .82)
-59 7 27 (736)	Left IFG pars opercularis, anterior Insula	.66 (.37, .81)
40 5 5 (597)	Right IFG pars opercularis, anterior Insula	.59 (.24, .77)
-31 -10 1 (190)	Left Putamen	.58 (.22, .77)
40 -12 59 (102)	Right M1	.61 (.28, .79)
54 1 43 (36)	Right M1, premotor cortex	.74 (.52, .86)
-17 -20 7 (17)	Left thalamus	.43 (-.06, .69)
-17 -10 19 (10)	Left thalamus	.71 (.46, .84)

ROI: region of interest; ICC: intraclass correlation coefficient; TIF: paced thumb-index finger tapping; TAF: paced thumb alternating finger opposition; TIF/TAFfast: unpaced condition with movement as fast as possible;
M1: primary motor cortex; S1: primary sensory cortex; SII: secondary sensory cortex; SMA: supplementary motor area; IFG: inferior frontal gyrus; IPL: inferior parietal lobule; SPL: superior parietal lobule; SMG: supramarginal gyrus; MCC: middle cingulate cortex.
Color-coding for ICC-values: red: 0 - .39 (poor); yellow: .40 - .59 (fair); light green: .60 - .75(good); dark green: >.75 (excellent)

Age Groups

Sample characteristics and performance

Table S4	Younger half (n = 16)		Older half (n = 15)	
	Baseline (Mean ± SD)	Follow-Up (Mean ± SD)	Baseline (Mean ± SD)	Follow-Up (Mean ± SD)
Age (years)	25.7 ± 4.2		46.3 ± 8.5	
Sex (n, % female)	10 (62.5)		6 (40)	
Education (years)	15.6 ± 3.2		16.8 ± 3.5	
TIFfast performance (Taps/second)	3.74 ± .90	3.83 ± .83	3.70 ± 1.43	3.88 ± 1.13
TAFfast performance (Taps/second)	2.91 ± .72	3.02 ± .68	3.11 ± 1.12	3.08 ± 1.06
TIF: Paced thumb-index finger tapping; TAF: paced thumb alternating finger opposition; TIF/TAFfast: unpaced condition with movement as fast as possible				

Dice similarity coefficients

Table S5 - Dice Similarity Coefficients (DSC)										
Condition	TIF		TIFfast		TAF		TAFfast		AllC	
Age group	young	old	young	old	young	old	young	old	young	old
Threshold p=.05	.347	.557	.567	.617	.573	.707	.685	.692	.740	.716
Threshold p=.001	.293	.571	.558	.580	.430	.641	.626	.662	.644	.665
Threshold p_{FWE}=.05	.081	0*	.462	.512	.145	.282	.385	.463	.444	.491
TIF: Paced thumb-index finger tapping; TAF: paced thumb alternating finger opposition; TIF/TAFfast: unpaced condition with movement as fast as possible; AllC: Contrast of all tapping conditions vs. all rest conditions; young: younger half of the study sample (n=16); old: older half of the study sample (n=15); 0*: no overlapping activation between sessions										

Intraclass correlation coefficients

Table S6 – Average ICC values per condition and age group

	TIF			TIFfast			TAF			TAFfast			AllC			mean of 5 conditions		
Age group	young	old	stat	young	old	stat	young	old	stat	young	old	stat	young	old	stat	young	old	stat
Literature	.40	.60	p=.017	.46	.66	p=.013	.47	.41	p=.49	.41	.67	p<.001	.54	.66	p=.10	.45	.60	p<.001
Anatomical	.39	.65	p=.002	.53	.75	p<.001	.37	.18	p=.020	.34	.70	p<.001	.60	.65	p=.41	.45	.59	p<.001
Conjunction	.43	.60	p=.20	.48	.62	p=.12	.45	.46	p=.93	.39	.58	p=.06	.60	.60	p=.97	.47	.57	p=.023

TIF: Paced thumb-index finger tapping; TAF: paced thumb alternating finger opposition; TIF/TAFfast: unpaced condition with movement as fast as possible; AllC: Contrast of all tapping conditions vs. all rest conditions; young: younger half of the study sample (n=16); old: older half of the study sample (n=15); stat: significance of the two-sample t-test comparing the older and younger half of the sample.

Figure S1: Depiction of the literature-based ROI-set. ROIs were created by drawing spheres with 10mm radius centered on peaks reported in an activation likelihood estimation[1]. Note the overlap of bilateral M1 and S1 ROIs (40% overlap).

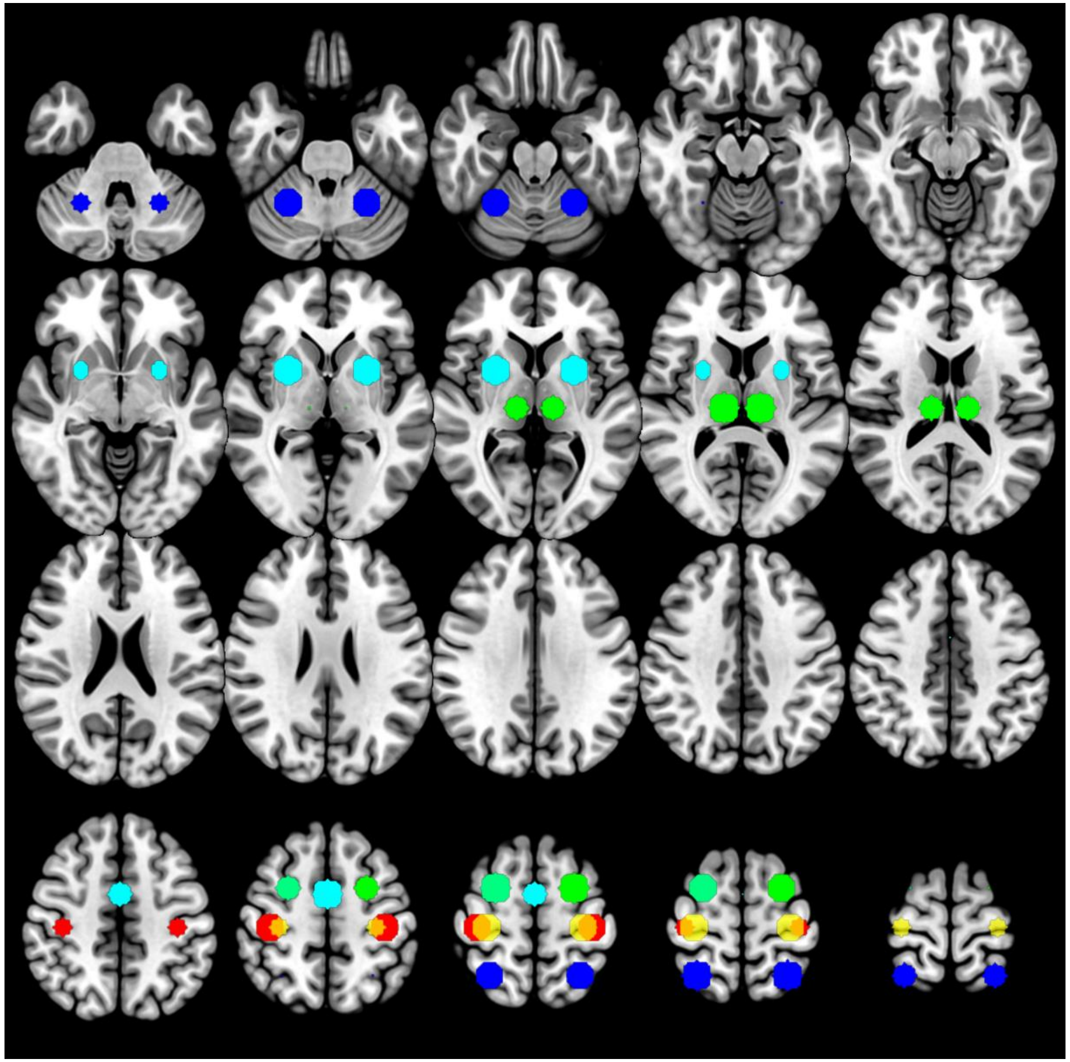

Figure S2: Depiction of the anatomical ROI-set from the AAL-atlas[2].

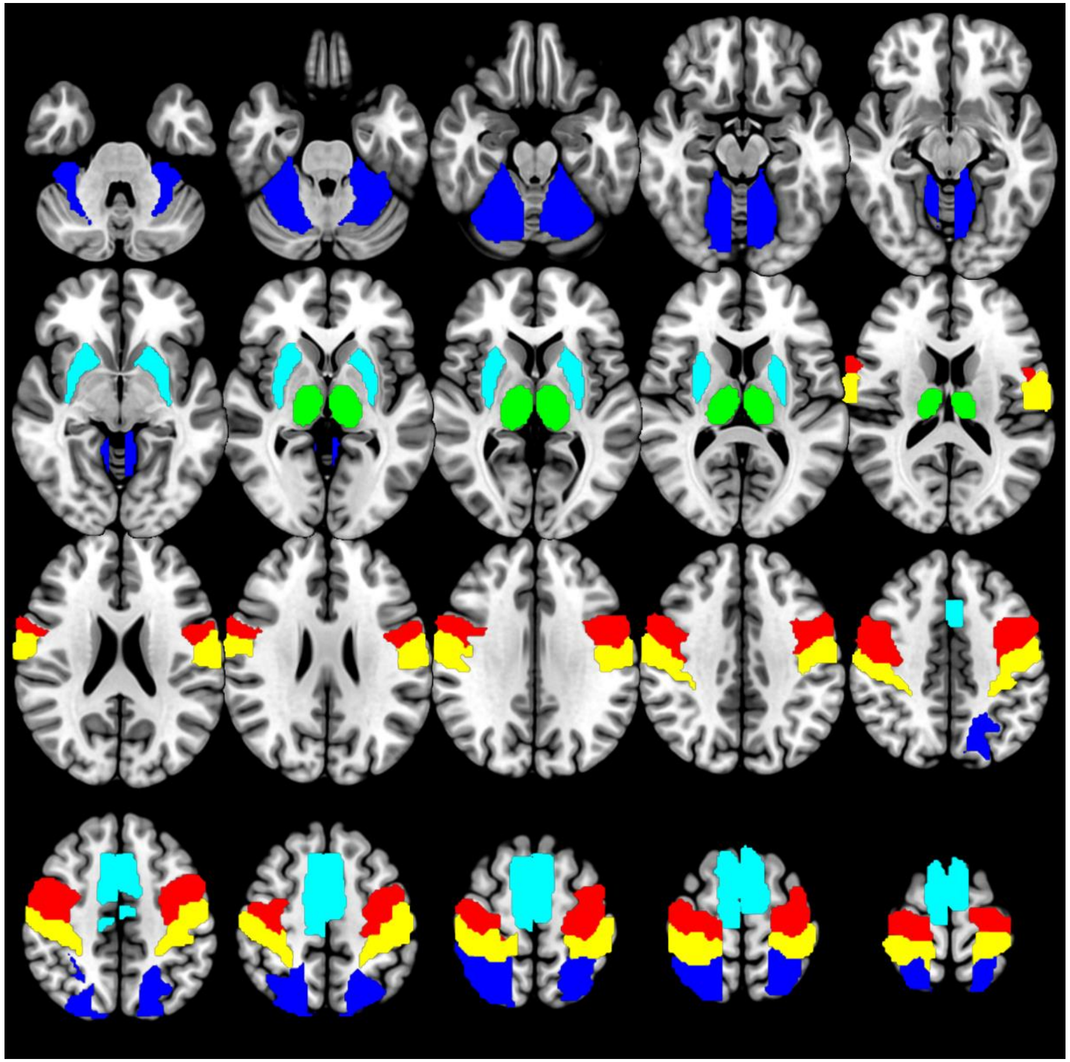

Figure S3: Depiction of ROIs derived from conjunction analysis of paced Thumb – Index Finger tapping vs. Listen contrast (TIF) at threshold $p_{FWE} < .05$.

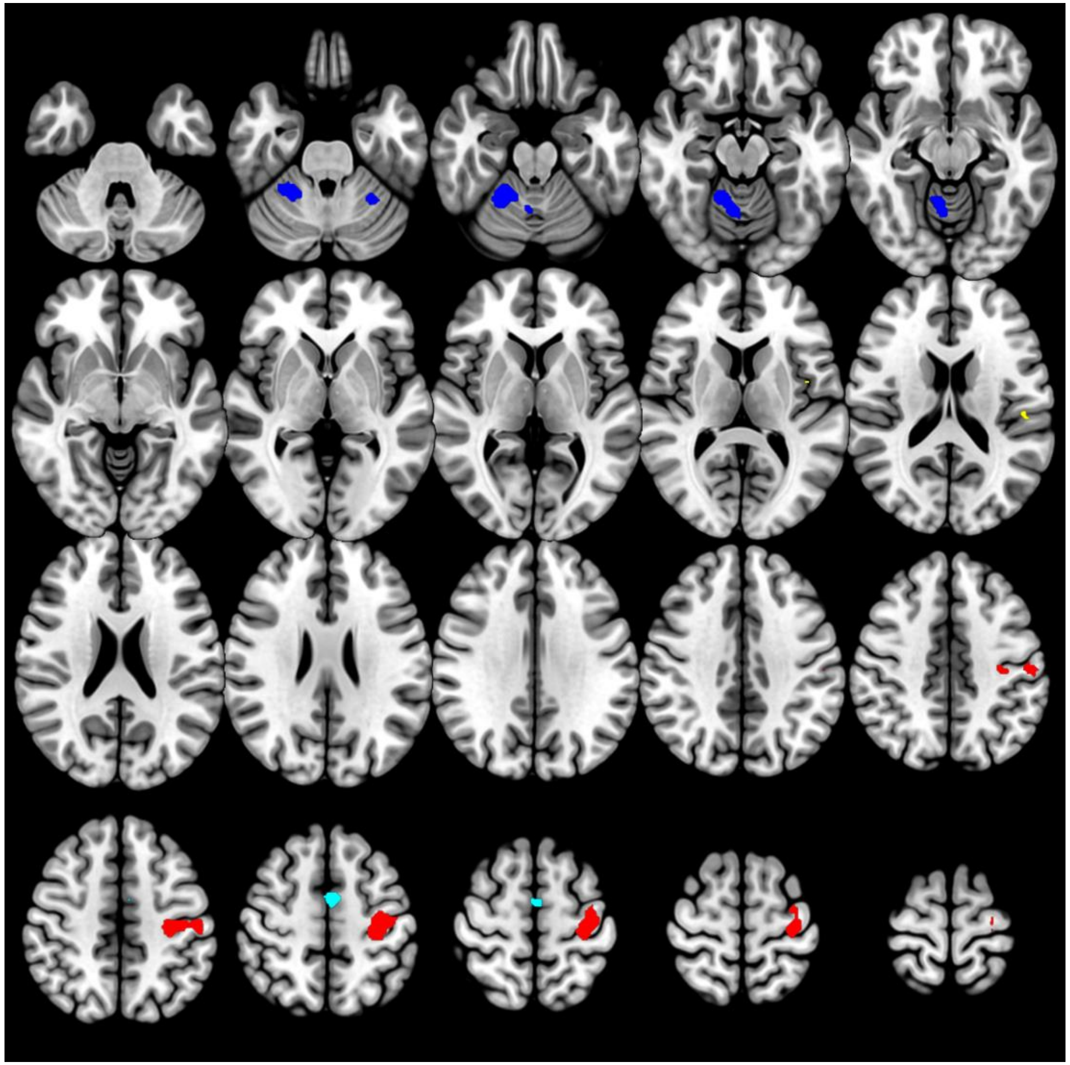

Figure S4: Depiction of ROIs derived from conjunction analysis of unpaced Thumb – Index Finger tapping vs. Rest contrast (TIFfast) at threshold $p_{FWE} < .05$.

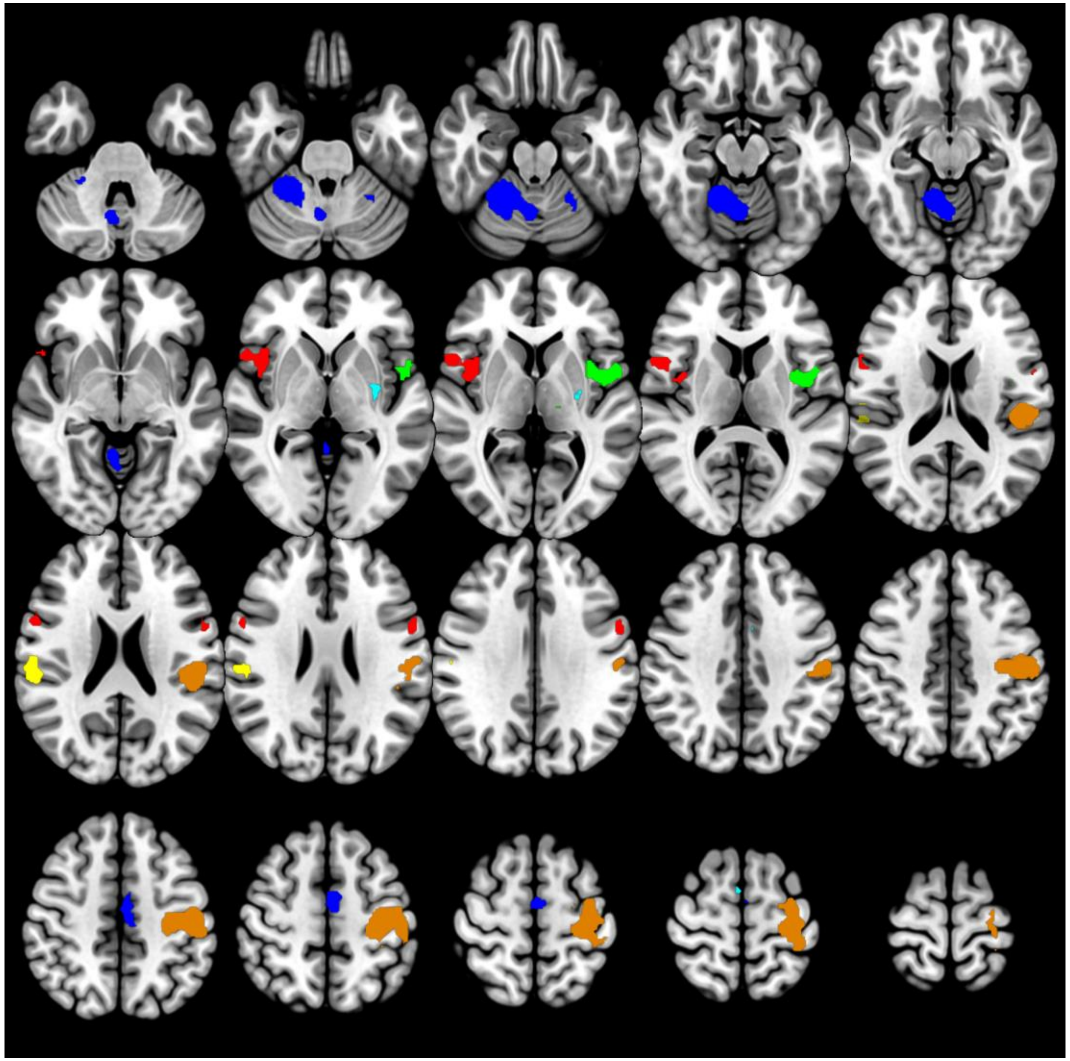

Figure S5: Depiction of ROIs derived from conjunction analysis of paced Thumb – Alternating Finger opposition vs. Listen contrast (TAF) at threshold $p_{FWE} < .05$.

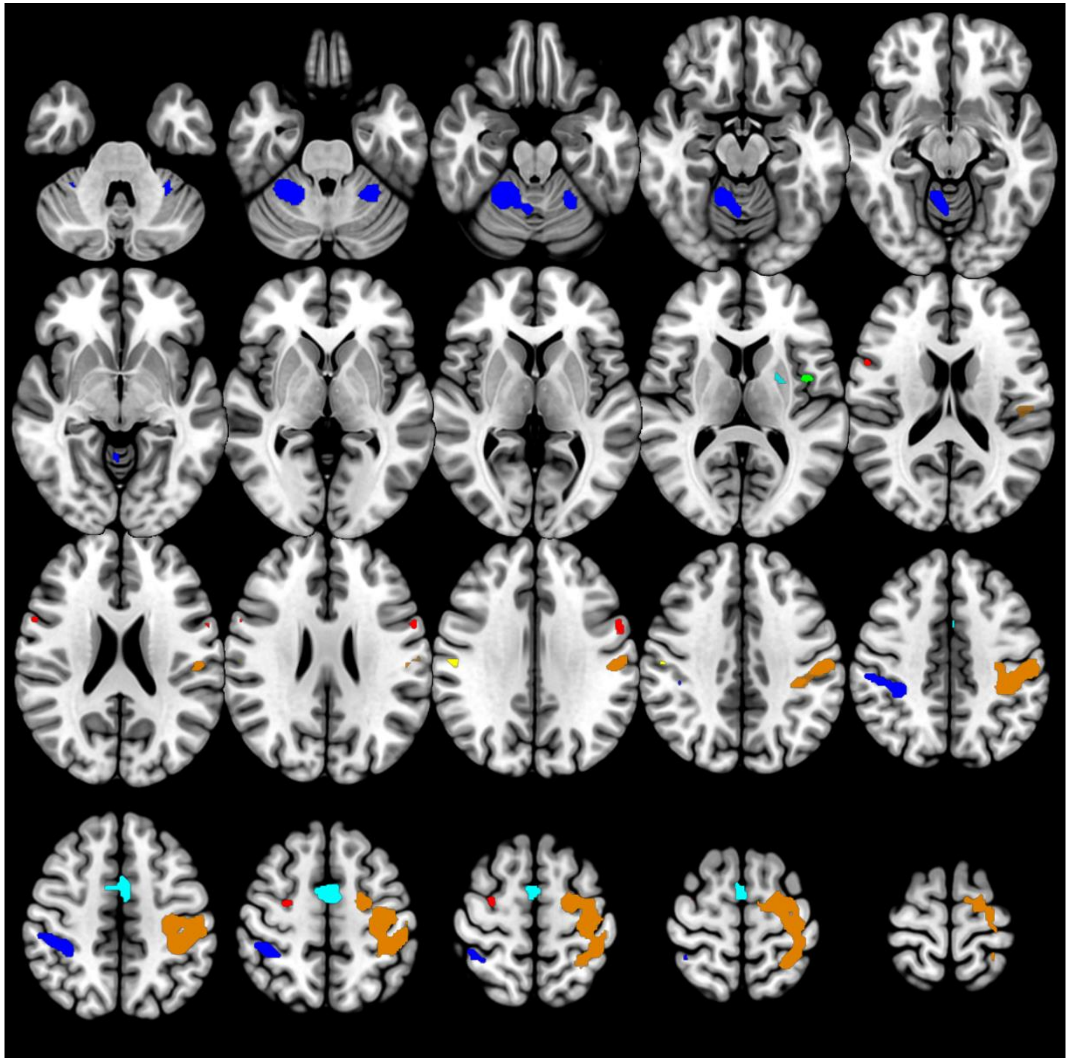

Figure S6: Depiction of ROIs derived from conjunction analysis of unpaced Thumb – Alternating Finger opposition vs. Rest contrast (TAFfast) at threshold $p_{FWE} < .05$.

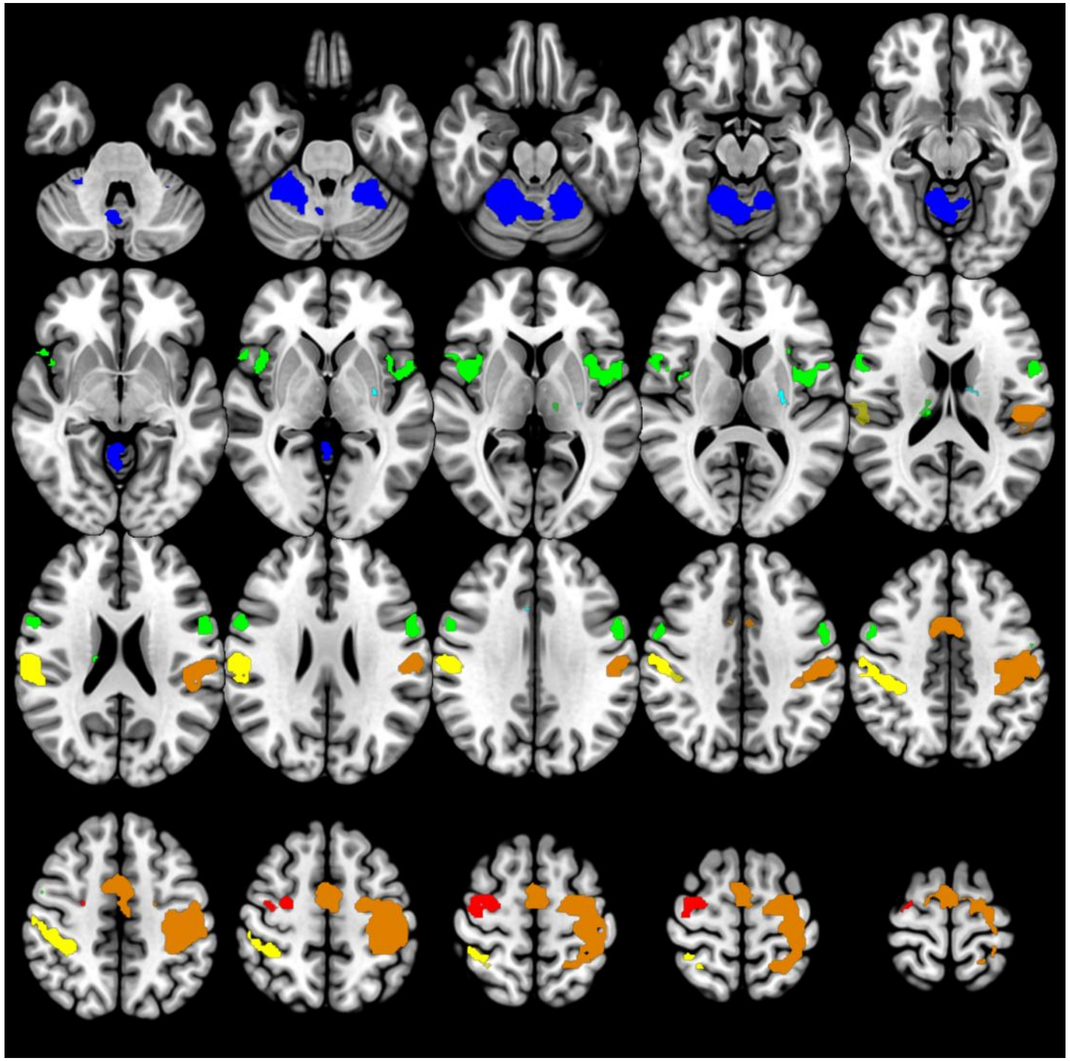

Figure S7: Depiction of ROIs derived from conjunction analysis of all tapping conditions vs. all rest conditions contrast (AllC) at threshold $p_{FWE} < .05$.

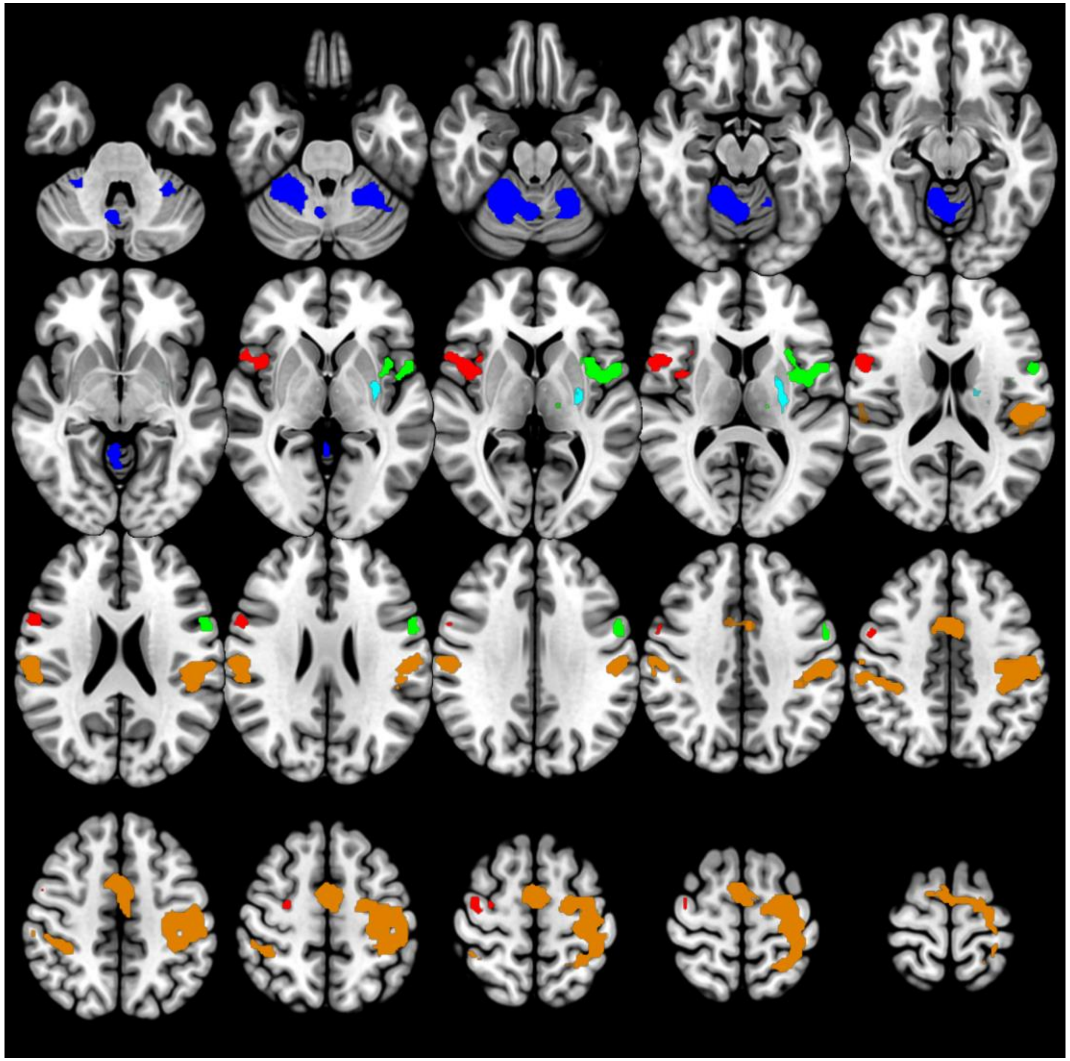

Figure S8: Overlay of ROI-sets. Red: Anatomical atlas ROIs; Yellow: Literature-based ROIs; Green: Example of conjunction analysis based ROIs (AllC) at threshold $p_{FWE} < .05$.

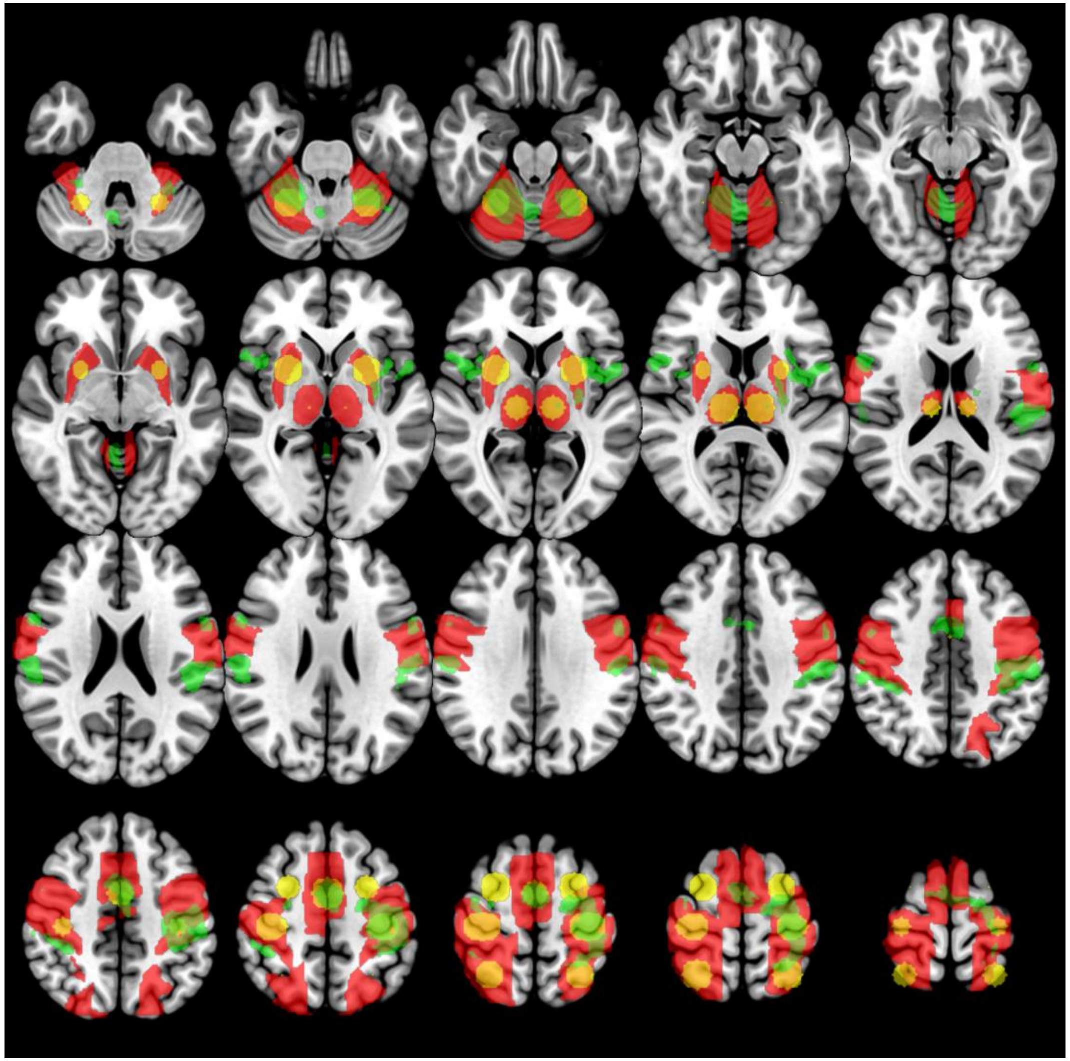

Figure S9: Side-by-side comparison of activation overlap by age group and contrast at threshold of p < .001. TIF: Paced thumb-index finger tapping; TAF: paced thumb alternating finger opposition; TIF/TAFfast: unpaced condition with movement as fast as possible; All: Contrast of all tapping conditions vs. all rest conditions; young: younger hal1f of the study sample (n=16); old: older half of the study sample (n=15); DSC: Dice similarity coefficient.

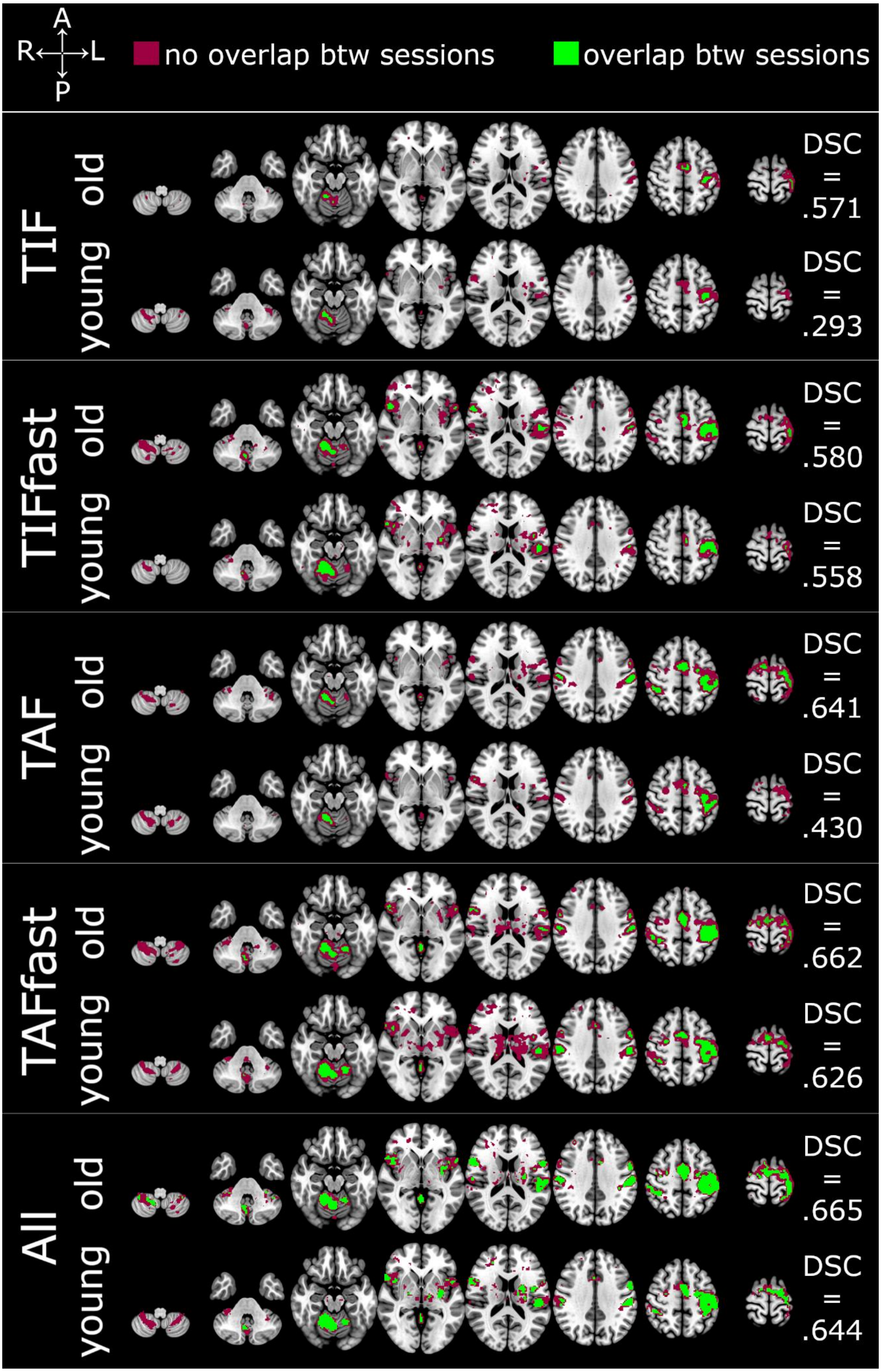

1. Hardwick, R.M., et al., *A quantitative meta-analysis and review of motor learning in the human brain.* Neuroimage, 2013. **67**: p. 283-97.
2. Tzourio-Mazoyer, N., et al., *Automated anatomical labeling of activations in SPM using a macroscopic anatomical parcellation of the MNI MRI single-subject brain.* Neuroimage, 2002. **15**(1): p. 273-89.
